# The GpIA7 effector from the potato cyst nematode *Globodera pallida* targets potato EBP1 and interferes with the plant cell cycle

**DOI:** 10.1093/jxb/erab353

**Published:** 2021-07-26

**Authors:** Mirela C Coke, Sophie Mantelin, Peter Thorpe, Catherine J Lilley, Kathryn M Wright, Daniel S Shaw, Adams Chande, John T Jones, Peter E Urwin

**Affiliations:** 1 Centre for Plant Sciences, University of Leeds, Leeds LS2 9JT, UK; 2 The James Hutton Institute, Dundee Effector Consortium , Invergowrie, Dundee DD2 5DA, UK; 3 School of Biology, University of St Andrews, North Haugh, St Andrews KY16 9TZ, UK; 4 Cardiff University, UK

**Keywords:** Cell cycle, cyclin D3;1, EBP1, endoreduplication, potato, RBR1, nematode

## Abstract

The potato cyst nematode *Globodera pallida* acquires all of its nutrients from an elaborate feeding site that it establishes in a host plant root. Normal development of the root cells is re-programmed in a process coordinated by secreted nematode effector proteins. The biological function of the *G. pallida* GpIA7 effector was investigated in this study. *GpIA7* is specifically expressed in the subventral pharyngeal glands of pre-parasitic stage nematodes. Ectopic expression of *GpIA7* in potato plants affected plant growth and development, suggesting a potential role for this effector in feeding site establishment. Potato plants overexpressing *GpIA7* were shorter, with reduced tuber weight and delayed flowering. We provide evidence that GpIA7 associates with the plant growth regulator StEBP1 (ErbB-3 epidermal growth factor receptor-binding protein 1). GpIA7 modulates the regulatory function of StEBP1, altering the expression level of downstream target genes, including *ribonucleotide reductase 2*, *cyclin D3;1*, and *retinoblastoma related 1*, which are down-regulated in plants overexpressing *GpIA7*. We provide an insight into the molecular mechanism used by the nematode to manipulate the host cell cycle and demonstrate that this may rely, at least in part, on hindering the function of host EBP1.

## Teaser

We report the first secreted nematode effector protein that can alter the expression of key host cell cycle components by modulating the regulatory function of a plant growth regulator.

## Introduction

The potato cyst nematode *Globodera pallida* is a sedentary endo-parasitic nematode that has a close biotrophic relationship with its host. Second-stage juveniles (J2s) of *G. pallida* hatch from eggs enclosed in cysts in response to host root exudates. Following chemical cues, the J2s locate a host root, penetrate the root close to the tip, and migrate intracellularly through the cortical cells, until they reach the inner cortex layers. Here, they become sedentary and each establishes a feeding site called a syncytium, by reprogramming the host cells. The syncytium is the sole source of nutrition for the nematode. The feeding site originates from an initial single cell, and neighbouring cells are progressively incorporated by cell wall dissolution, in a pre-defined pattern, via a ‘cortex bridge’ towards the vascular cylinder (Sobczak and Golinowski, 2011). Syncytial nuclei become enlarged and lobed soon after feeding site induction, and it has been suggested that they undergo endoreduplication (Golinowski *et al.*, 1996).

These elaborate changes in plant cells that culminate in the formation of the syncytial feeding site are thought to be orchestrated by effector proteins from the nematode secretions, which are mainly produced in the pharyngeal glands of the nematode and injected into the selected host cell through the stylet. Studies that have identified nematode effectors and predicted effectoromes (e.g. Bellafiore *et al.*, 2008; Maier *et al.*, 2013; Thorpe *et al.*, 2014) revealed that the majority of effectors are pioneers with no sequence similarity to known proteins. Therefore, their functions in the plant cell cannot be predicted and our knowledge of the molecular mechanisms used by the nematode to re-route the cells’ normal growth and development processes is fragmented.

Changes leading to the formation of the syncytium require the nematode to manipulate progression of the cell cycle in the initial syncytial cell (Sobczak and Golinowski, 2011). Cell cycle progression is strictly controlled by an evolutionarily conserved mechanism that relies on distinct combinations of cyclin-dependent kinases (CDKs) and cyclins (CYCs) to phosphorylate downstream targets (De Veylder *et al.*, 2007). In syncytia, the normal cell cycle status is diverted towards the onset of the endocycle (de Almeida Engler and Gheysen, 2013). This is a variant of the cell cycle where the mitosis phase is bypassed entirely through modulation of CDK/CYC activity (Gutierrez, 2009). Key cycle genes (*CDKA1;1*, *CDKB1;1*, *CYCB1;1*, and *CYCA2;1*) are up-regulated early in syncytial development and, with the exception of *CDKA1;1*, are strongly down-regulated by 9 days post-infection (dpi) (de Almeida Engler *et al.*, 1999). Despite extensive evidence provided by expression analysis and cytological studies, no nematode effectors targeting the core plant cell cycle machinery have been identified yet (de Almeida Engler and Gheysen, 2013). Thus, the molecular mechanism used by the nematode to re-programme the cell cycle remains unknown.

The *GpIA7* effector gene was previously identified in *G. pallida* as a pioneer sequence unique to the cyst nematode parasitome (Grenier *et al.*, 2002). The gene encoding this effector is expressed in the subventral glands of *G. pallida* and the predicted protein has one ShK domain (Blanchard *et al.*, 2007). This domain is abundantly represented in the *Caenorhabditis elegans* proteome where ShK-like toxins are part of the so-called core set of immune effectors that are induced in response to invasion of *C. elegans* by diverse pathogens (Shivers *et al.*, 2008; Simonsen *et al.*, 2012), but the functional role of this domain in the defence response of the nematode is unknown. The Hs-Tyr effector of the beet cyst nematode *Heterodera schachtii*, which contains four ShK domain-like sequences, triggers changes in plant hormone homeostasis (Habash *et al.*, 2017). However, Hs-Tyr shares no overall sequence similarity with GpIA7 and only 16% identity in one ShK domain, suggesting that they have different functions.

Plant EBP1 proteins are structural and functional homologues of human EBP1 (ErbB-3 epidermal growth factor receptor-binding protein 1) (Horváth *et al*., 2006), which is a DNA/RNA- and protein-binding protein with a role in cell proliferation, differentiation, and apoptosis (Yoo *et al.*, 2000; Zhang *et al.*, 2002; Liu *et al.*, 2006; Okada *et al.*, 2007; Figeac *et al.*, 2014). The potato *StEBP1* gene encodes a 387 amino acid protein that regulates organ size through cell growth and proliferation, and whose levels are influenced by auxin (Horváth *et al*., 2006). Ectopic expression of *EBP1* in transgenic plants increases or reduces organ growth in a dose-dependent manner and alters the timing of the vegetative to reproductive transition (Horváth *et al*., 2006; Cheng *et al.*, 2016). EBP1 is a target for the highly conserved regulator TOR (target of rapamycin) kinase (Deprost *et al.*, 2007) and regulates translation in addition to counteracting *retinoblastoma related* (*RBR*) switch to differentiation in root meristems (Lokdarshi *et al.*, 2020). It has been shown to maintain root meristem activity and growth in sugar-limited conditions (Lokdarshi *et al.*, 2020), supporting its role in regulation of abiotic stress resistance in plants (Cao *et al.*, 2008; Cheng *et al.*, 2016).

In this study, we investigated the biological function of the GpIA7 effector. GpIA7 interacts with the potato host StEBP1, and its ectopic production in potato plants results in reduced expression of *StRNR2* (*ribonucleotide reductase 2*), *StCYCD3;1* (*cyclin D3;1*), and *StRBR1* (*retinoblastoma related 1*), which are key cell cycle components under the regulation of StEBP1. These alterations in expression of cell cycle genes correlate with phenotypic changes observed in *GpIA7*-overexpressing plants. We hypothesize that the GpIA7 effector interferes with the function of EBP1, an auxin-induced protein that modulates key cell cycle components, as part of the molecular mechanism used by the nematode to manipulate the cell cycle and initiate endoreduplication.

## Materials and methods

### Cloning *GpIA7* from *G. pallida* and *StEBP1* from potato

The *GpIA7* coding region, lacking the predicted signal peptide, was amplified by PCR from cDNA generated from J2 stage *G. pallida* (population Lindley, pathotype Pa2/3) as described previously (Thorpe *et al.*, 2014), using gene-specific primers (for GPLIN_000638300) that included a leader sequence (ACCATG) for translation (see Supplementary Table S1) and the proof reading KOD DNA polymerase (Novagen), according to the manufacturer’s instructions. The amplified product was purified from a 1.5% (w/v) agarose gel, using the QIAquick Gel Extraction Kit (Qiagen), and inserted by T/A cloning into the pCR8/GW/TOPO Gateway ENTRY vector (Life Technologies). The effector clone was subsequently transferred into the pGBKT7 vector, in-frame with the GAL4-binding domain using the In-fusion cloning system (Clontech) for yeast two-hybrid (Y2H) screening, or recombined into a series of Gateway-compatible binary expression vectors according to the LR clonase protocol (Life Technologies): pK7WG2 for potato transformation, pK7WGF2/pH7WGR2 eGFP/mRFP (enhanced green fluorescent protein/monomeric red fluorescent protein) N-terminal fusions (Karimi *et al.*, 2002), and the pCL113 vector [yellow fluorescent protein C-terminal domain (YFPc); Bos *et al.*, 2010] to create an N-terminal split-YFP fusion for bimolecular fluorescence complementation (BiFC) assays. The *Gp4D06* and *Gp16H02* coding regions, used as a control bait for Y2H screening, were amplified from *G. pallida* cDNA and subsequently transferred into the pGBKT7 vector as described above.

For cloning of *StEBP1*, total RNAs were isolated from potato roots (*Solanum tuberosum* cv. Désirée) using an RNeasy Mini Kit (Qiagen). cDNA was generated using Superscript II reverse transcriptase (Life Technologies) with poly(dT) primers, following the manufacturer’s instructions. The complete *StEBP1* coding region (Supplementary Fig. S1A) was amplified using gene-specific primers [Supplementary Table S1; based on the GenBank sequence NM_001288259 (Horváth *et al*., 2006), corresponding to the potato gene locus PGSC0003DMG400030365]. It was inserted into the pCR8/GW/TOPO Gateway ENTRY vector and transferred to expression vectors as described above, only into pH7WGR2, the split-YFP vector pCL112 (YFPn domain; Bos *et al.*, 2010), and the prey vector pGADT7-Rec (Clontech) to generate a fusion with the GAL4 activation domain for the Y2H assay.

The integrity of all clones was checked by sequencing. The binary vectors were transformed into *Agrobacterium tumefaciens* strain GV3101 harbouring the helper plasmid pBBR1MCS5-VIRG-N54D that carries the virG^N54D^ virulence factor (van der Fits *et al.*, 2000) for transient expression in *Nicotiana benthamiana* or into *A. tumefaciens* strain LBA4404 harbouring the same plasmid for transformation of potato.

### Generation and phenotypic analysis of transgenic potato lines expressing *GpIA7*.

Transformation of potato cv. Désirée with pK7WG2:*GpIA7* using an *A. tumefaciens* co-cultivation method (Bajroviç *et al.*, 1995) generated 11 independent transgenic lines. Mock-transformed control lines underwent the same regeneration steps but in the absence of *Agrobacterium* and antibiotic selection. The expression of *GpIA7* in the regenerated potato lines was evaluated by quantitative reverse transcription–PCR (qRT–PCR).

Phenotypic analysis was performed on three *GpIA7*-expressing potato lines and one control line growing in a glasshouse at 21 °C under a 16 h photoperiod. Eight plants of each line were taken from tissue culture and grown in a mix of potting compost (Sinclair Horticulture) and Perlite (~20:1 ratio) in 7 cm pots. After 4 weeks, plants were transferred to potting compost in 18 cm pots positioned according to a Latin square design to reduce the impact of local environmental factors. Measurements of plant height, length, and width of a selected terminal leaflet (youngest leaf >1 cm at the start of the measurements) were recorded periodically from this point. The number of days to complete opening of the first flower was recorded. The number and fresh weight of tubers, number of leaf nodes on the main stem, and fresh and dry above-ground plant weight were recorded at maturity, at the start of natural senescence.

### Yeast two-hybrid screening

The Matchmaker Gold Y2H system (Clontech) was used to screen a potato root cDNA library generated using d(T) primers, in yeast cells, with GpIA7 as a bait. Root tips of potato cv. Désirée plantlets in growth pouches (Mega International) were infected with ~175 *G. pallida* J2s per plant. Root tissue was harvested at 1–6 dpi and pooled for extraction of total RNA using the RNeasy mini kit (Qiagen). A cDNA library of >1 million independent clones was generated in the pGADT7-Rec vector as a fusion with the GAL4 activation domain and transformed into *Saccharomyces cerevisiae* Y187 strain. The pGBKT7-GpIA7 bait clone was transformed into the mating-compatible *S. cerevisiae* Gold strain and tested for absence of autoactivation of the reporter genes.

The screening was performed according to the Matchmaker Gold kit manual (Clontech) with the following modifications. The ratio between bait and prey cells for the mating was reduced to 2:1 to account for differences in the growth rate between bait and prey. The total number of yeast cells used per screening was reduced to 6×10^7^ cells and the mating volume was reduced to 25 ml. Following the screening process, the prey sequences were selectively amplified by PCR performed on Zymolase-treated diploid colonies using Matchmaker Insert Check PCR Mix 2 (Clontech). PCR products were purified (Qiaquick PCR purification kit, Qiagen) and directly sequenced using T7 primer.

To test selected interactions for specificity, the bait and prey clones rescued from the yeast diploid cells were re-introduced into yeast single strains, mated, and the diploids isolated on low stringency selection plates and further tested on high stringency selection.

### *In planta* subcellular localization and bimolecular fluorescence complementation assay

For subcellular localization *in planta*, the eGFP::GpIA7 and mRFP::StEBP1 fusion protein constructs were transiently expressed individually or together, using agroinfiltration with *A. tumefaciens* GV3101 (bacteria at OD_600nm_ 0.02–0.1), in the leaves of 4-week-old *N. benthamiana* plants grown in a glasshouse at 21 °C, as described in Thorpe *et al.* (2014). Free eGFP was used as a control (Mei *et al.*, 2015). For BiFC analysis, the YFPc::GpIA7 and YFPn::StEBP1 constructs were used, as well as the positive interactor pair YFPc::StBSL1/YFPn::PiAVR2 cloned in the same vectors (Saunders *et al.*, 2012).

The fluorescence signals were imaged 48 h post-inoculation using a Zeiss LSM 710 confocal microscope. The eGFP fluorescence was imaged with excitation/emission wavelengths (λ) of λ488 nm/λ495–530 nm. The excitation/emission for mRFP was at λ561 nm/λ592–631 nm, and for YFP at λ514 nm/ λ530–575 nm. Autofluorescence from chlorophyll generated by excitation at these wavelengths was collected at λ657–737 nm.

### Pull-down assay

The coding region of *GpIA7* lacking its signal peptide region was cloned in-frame with an N-terminal 6×His tag in the pQE30 bacterial expression vector (Qiagen) and transformed into *Escherichia coli* strain M15(pREP4). A 100 ml bacteria culture (OD_600nm_ 0.5) was induced with 2 mM isopropyl-β-d-1-thiogalactopyranoside (IPTG) for 3 h at 30 °C and cells were harvested and re-suspended in sonication buffer (50 mM phosphate buffer, 300 mM NaCl, pH 8.0 with 100 µg ml^−1^ lysozyme). The soluble lysate containing 6×His-GpIA7 fusion protein was incubated with 100 µl of Ni-NTA resin for 1 h on ice, followed by removal of the lysate by centrifugation (3000 *g*, 3 min, at room temperature). The Ni-NTA resin was re-suspended in wash buffer (50 mM phosphate buffer, 500 mM NaCl, 40 mM imidazole pH 8.0). The control resin underwent a mock procedure.

The coding region of *StEBP1* (full length) was cloned in-frame with an N-terminal 3×FLAG tag that had been introduced into pBI121 (Clontech) from which the β-glucuronidase (GUS) gene had been removed and replaced by a *Kpn*I restriction site. The mature coding region of an unrelated gene (*GpaGS22*; Lilley *et al.*, 2018) cloned into the same vector was used as a control. The resulting constructs were transformed into *A. tumefaciens* GV3101 for transient expression in *N. benthamiana* leaves as already described. Total soluble protein was extracted from agro-infiltrated leaves 2 d after infiltration and prepared as described by Xu *et al.* (2015) except that EDTA and DTT were omitted from the extraction buffer, which also included cOmplete™ Protease Inhibitor Cocktail (Roche). The cleared cell lysates were filtered through a 0.45 µm filter and used immediately in pull-down assays.

To perform the pull-down assay, 10 µl (~19 µg of GpIA7) of Ni-NTA resin (± bound GpIA7) was incubated with 250 µl of plant cell lysate for 2 h on ice with gentle regular mixing. The resin was washed five times with extraction buffer containing 40 mM imidazole. Bound proteins were eluted with elution buffer (50 mM NaH_2_PO_4_, 500 mM NaCl, 250 mM imidazole, pH 6.0) and resolved alongside input samples (bacterial or plant lysate) on 4–12% Bis-Tris gels (Bio-Rad). Subsequently they were transferred to a 0.45 µm Invitrolon PVDF membrane (Invitrogen). The membranes were immunoblotted with primary antibodies: Penta-His (1:1000, Qiagen) or anti-Flag M2 (1:2000, Sigma) followed by incubation with anti-mouse IgG alkaline phosphatase-conjugated secondary antibody (1:10 000, Sigma). The proteins were detected with SigmaFast NBT/BCIP.

### Generation of *StEBP1* RNAi potato plants

For generation of the *StEBP1* silencing construct, a 475 bp fragment of the *StEBP1* cDNA clone was amplified in two separate PCRs, with two sets of primers containing restriction sites (Supplementary Table S1) such that it could be cloned into the pHannibal vector (Wesley *et al.*, 2001) in sense (*Xho*I/*Kpn*I) and antisense (*Xba*I/*Cla*I) orientations, to form an inverted repeat construct under control of the *Cauliflower mosaic virus* (CaMV) 35S promoter. The complete expression cassette was transferred to pART27 (Gleave, 1992) and the resulting construct was introduced into *A. tumefaciens* LBA4404 for transformation of potato cv. Désirée as described above. The level of *StEBP1* silencing in the regenerated lines was evaluated by qRT–PCR. Total RNA was isolated from leaves of 5-week-old *in vitro* cultured potato plants using an RNeasy Plant Mini kit (Qiagen). cDNA was generated from 500 ng of DNase-treated RNA using the iScript cDNA synthesis kit (Bio-Rad). The quantification of gene expression was carried out using SsoAdvanced™ Universal SYBR^®^ Green Supermix (BioRad) and a CFX Connect instrument (BioRad, CA, USA), with primers detailed in Supplementary Table S1. The potato elongation factor 1α gene (*StEF1α*) was used for normalization. Primer pair efficiencies and Ct values were determined using the BioRad CFX Manager 3.1 software. Relative expression of *StEBP1* in control and transgenic RNAi potato lines was determined using the 2^(ΔΔCt)^ method. Each reaction was carried out in triplicate, and expression analysis of the selected lines was carried out on two separate occasions.

### RNAi of GpIA7 in *Globodera pallida* J2s

A 143 bp fragment of the *GpIA7* coding region was amplified (see Supplementary Table S1, for primers) and cloned into the L4440 vector (Addgene), using the *Xba*I and *Xho*I restriction enzyme sites included in the primers. The resulting plasmid, harbouring the *GpIA7* fragment cloned between two T7 promoters, was used to generate dsRNAs with the Megascript RNAi kit following the manufacturer’s instructions (Ambion). The integrity of the dsRNAs was checked on an agarose gel and the concentration was estimated using a NanoDrop ND-1000 spectrophotometer (NanoDrop Technologies).

The dsRNAs were delivered by incubating 2000–3000 J2s of *G. pallida* overnight in a solution of 200 ng µl^–1^ dsRNA, 50 mM octopamine, and 3 mM spermidine in M9 buffer (Urwin *et al.*, 2002). *Globodera pallida* J2s incubated in soaking solution without dsRNA or treated with GFP dsRNAs acted as controls. To check for the down-regulation of *GpIA7* transcripts in the treated J2s, qRT–PCR was performed on an equal number of J2s removed from each treatment.

### Expression analysis of cell cycle-related genes in potato

The expression levels of *StCYCD3;1*, *StCDKBA;1*, *StRBR1*, and *StRNR2* genes were analysed by qRT–PCR in three *GpIA7* overexpression lines, while expression of the *StRBR1* gene was analysed in three *StEBP1* (RNAi) lines.

Total RNA was isolated from transgenic potato lines and equivalent wild-type material as described above, and cDNA for qRT–PCR was generated from 1 µg of RNA. *StEF1-α* (Nicot *et al.*, 2005) was used for normalization. The expression of *StEF1*-*α* was confirmed to be stable across treatments, validating its use as a reference gene (see Supplementary Fig. S2). Primers used are listed in Supplementary Table S1. Two technical and three biological replicates were carried out for each line. Data analysis and relative quantification of target gene expression were carried out as described above.

### Nematode culture and infection assays

Cysts of *G. pallida* population Lindley (Pa2/3) were extracted from soil and exposed to potato root diffusate, at 20 °C, to stimulate hatching. J2s were collected each second day, washed, and kept in water at 10 °C until use. For nematode infection assay, 10 rooted plants for each potato line were transferred from *in vitro* culture to growth pouches (Mega International) and maintained at 21 °C with a 16:8 h light:dark cycle. After 2 d, five root tips per root system were each inoculated with 35 J2 nematodes. At 17 dpi, the roots were stained with acid fuchsin. The number of nematodes and their developmental stages were recorded for each plant.

For susceptibility tests of the StEBP1 (RNAi) potato lines, 11 rooted plantlets for each line were transferred from *in vitro* liquid culture to potting compost (Sinclair Horticulture). After 3 weeks, excess compost was removed from the root systems and the plants were transferred to 18 cm pots each containing 2.5 kg of 50:50 mix of sand:loam soil infected with 30 cysts of *G. pallida* per pot. After 12 weeks of growth, watering was withheld, and developed cysts were subsequently isolated by flotation from duplicate 100 g aliquots of well-mixed dried soil per pot using a Fenwick can. The number of cysts and their enclosed eggs g^−1^ soil were determined for each plant.

## Results

### *GpIA7* is expressed in the early developmental stages of *G. pallida*

Two paralogues of the *GpIA7* effector (GPLIN_000638300 and GPLIN_000740500) (see Supplementary Fig. S1C) were identified in the predicted gene complement of the annotated *G. pallida* genome assembly (Cotton *et al.*, 2014). Each encodes a protein of 73 amino acids with a predicted N-terminal signal peptide of 25 amino acids (Supplementary Fig. S1D). The calculated molecular weight of GpIA7 is 8.24 kDa with the signal peptide and 5.35 kDa without. The paralogues share 96% amino acid identity and the variable sites are located exclusively in the mature protein. The *GpIA7* clone isolated and characterized in this study (Supplementary Fig. S1C, D) is most similar to GPLIN_000638300, with only a single amino acid difference from the published genome sequence, and it will be referred to as *GpIA7*.

The expression of *GpIA7* was localized by *in situ* hybridization with a probe that would detect both *GpIA7* paralogues in the subventral glands of the pre-parasitic J2 (see Supplementary Fig. S1E) of *G. pallida*, confirming a previous report (Blanchard *et al.*, 2007). The temporal expression profiles of the two *GpIA7* paralogues (Supplementary Fig. S1F) were generated from normalized RNAseq data from eight developmental stages of *G. pallida* (Cotton *et al.*, 2014). *GpIA7* (GPLIN_000638300) is expressed in encysted eggs prior to hatching, but that expression increases >20-fold in pre-parasitic J2s. There is no detectable expression in the late parasitic stages analysed for that dataset, but there is a low level in adult males. This expression pattern is typical for many *G. pallida* effectors and falls within the cluster I expression type described by Thorpe *et al.* (2014). The GPLIN_000740500 paralogue displays a similar pattern, although some expression is maintained until 7 dpi, suggesting that *GpIA7* transcript might peak around the moment of infection or syncytium induction, then decrease over several days. This temporal expression profile was confirmed by qRT–PCR analysis, which shows that the expression of the GpIA7 paralogues is highly induced in J2s and remains high in parasitic nematodes established in roots at 5 dpi (Supplementary Fig. S1G). *GpIA7* proved to be refractory to RNAi by soaking J2 nematodes in dsRNA that targeted transcripts from both paralogues; no reduction in transcript abundance was detected by qRT–PCR on multiple experimental occasions (Supplementary Fig. S3).

### GpIA7 localizes *in planta* to the cytoplasm and in the nucleus

**Fig. 1.**
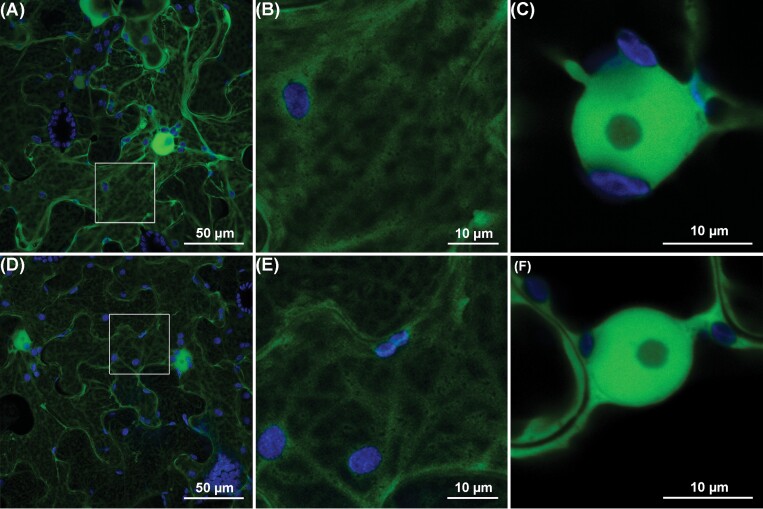
Subcellular localization of the GpIA7 effector *in planta*. *Agrobacterium*-mediated transient expression of (A–C) *GpIA7* lacking its endogenous signal peptide and tagged with the enhanced green fluorescent protein (eGFP) as a *eGFP::GpIA7* fusion in *Nicotiana benthamiana* leaves; (D–F) free eGFP protein. Pictures were taken 2 d post-infiltration by confocal microscopy, with GFP and autofluorescence from chloroplasts displayed in green and blue, respectively. (A, D) Maximum intensity projection images; (B, E) regions presented at higher magnification; and (C, F) images of single sections taken through the nucleus. Each localization experiment was replicated at least twice.

Effectors secreted into a plant cell from the oesophageal glands of the nematode can target different cellular compartments either alone or by interacting with host factors (Elling and Jones, 2014). Transient overexpression of GpIA7, lacking its endogenous signal peptide and tagged with eGFP, in the leaves of *N. benthamiana* revealed that GpIA7 is present in the cytoplasm and nucleoplasm of the plant cells, although it appears to be excluded from the nucleolus (Fig. 1A–C). This localization is similar to that of free eGFP (Fig. 1D–F) and is consistent with the fact that the *GpIA7* gene encodes a mature protein of only 5.35 kDa. GpIA7 has no nuclear or other localization motifs but is small enough for the fusion protein, and therefore the native effector, to diffuse into the nucleus.

### Potato plants overexpressing *GpIA7* are affected in growth and development but are not more susceptible to *G. pallida*

We generated potato lines overexpressing *GpIA7* in the *G. pallida*-susceptible cv. Désirée. We evaluated the impact of the transgene, in the absence of nematode infection, on the host plant growth and development, compared with a mock-transformed line (control). Three transgenic lines (GpIA7 4c, 5c, and 6c) were selected for the phenotypic analysis to span a range of *GpIA7* expression levels as judged by semi-quantitative RT–PCR.

**Fig. 2.**
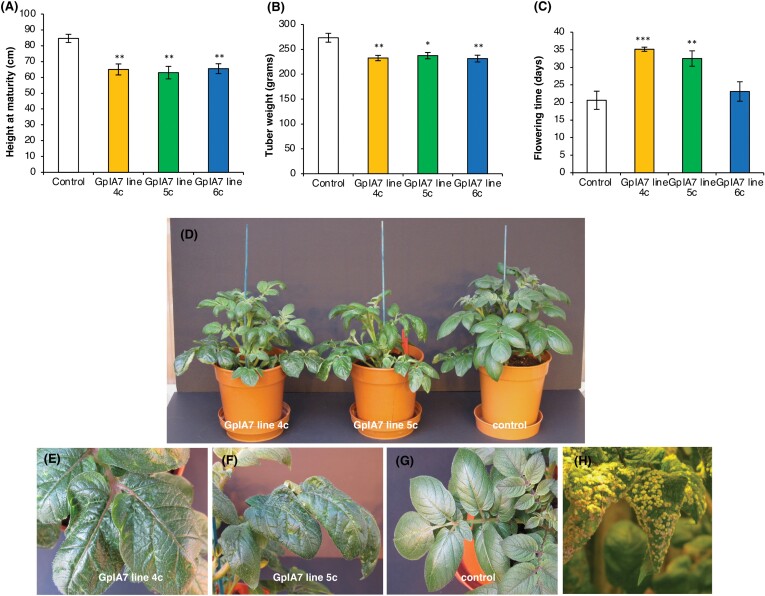
Phenotypic analysis of potato lines ectopically expressing *GpIA7*. Control lines are wild-type cv. Désirée plants that have been through equivalent tissue culture procedures. (A) The final height and (B) the total tuber weight per plant of all GpIA7 lines is significantly reduced compared with the control plants. (C) The flowering time, measured as the number of days from potting to the moment when the first flower opened, was significantly delayed in GpIA7 4c and GpIA7 5c lines, but not in GpIA7 line 6c, when compared with the control plants. (D) Ectopic expression of *GpIA7* in potato plants significantly alters the growth and the leaf morphology of the transgenic lines. (E–G) Close-up images of leaves of GpIA7 lines 4c and 5c, and control plants. GpIA7 lines display visible changes in leaf morphology as the leaf lamella of GpIA7 4c and GpIA7 5c lines shows a rugose, crinkly appearance with edges curling downwards or upwards, while the control plants have a smooth lamella; the altered leaf morphology is consistent across different plants of the same line. The plants shown have been grown for 6 weeks in the glasshouse. (H) Extensive intumescence observed on the leaves of the GpIA7 lines. Values are means and error bars represent the SE (*n*=8) with statistical differences indicated relative to control for *P<0.05, **P<0.01, or ***P<0.001 (ANOVA, followed by Bonferroni post-hoc correction).

Plants from all three GpIA7 potato lines grew more slowly than the control plants and were significantly shorter at maturity (Fig. 2A). The total fresh tuber weight in all GpIA7 potato lines was reduced compared with the control line (Fig. 2B). Ectopic expression of *GpIA7* also affected plant development as GpIA7 lines 4c and 5c flowered later than the control plants (Fig. 2C). In addition, the leaf morphology of the GpIA7 lines was visibly altered, with leaves of lines 4c and 5c displaying a rugose lamella with curling edges, while the control plants showed a smooth lamella and no curling (Fig. 2D–G). This morphological characteristic was consistent between plants (Fig. 2D–G). None of the other parameters measured (see the Materials and methods) differed significantly between transgenic and control plants. The phenotypic differences observed in the GpIA7 lines compared with the control are related to both plant growth and development processes.

Intriguingly, the GpIA7 plants showed severe symptoms of intumescence, which is characterized by 1–3 mm callus-like (Krumins *et al.*, 2015) protrusions on the leaves (Fig. 2H). The condition is a common physiological disorder that frequently affects *Solanaceae* plants grown in a controlled environment. Although all potato lines were positioned in the glasshouse according to the Latin square principle, these severe symptoms were observed only on the GpIA7 lines, while the control plants, and many transgenic lines within the same trial expressing unrelated *G. pallida* effectors, exhibited mild or no symptoms (unpublished data). The same phenomenon has been observed consistently (>6 occasions) whenever the GpIA7 lines have been grown in the glasshouse. This strongly suggests that overexpression of *GpIA7* not only impacts plant growth and development but also affects the plant’s ability to respond appropriately to environmental stimuli, thereby exacerbating the intumescence phenotype.

To determine if the ectopic expression of *GpIA7* alters the susceptibility of host plants to *G. pallida*, three transgenic potato lines were infected with *G. pallida* J2s in a pouch growth system. At 17 dpi there was no significant difference in the number or developmental stages of the nematodes present in roots of the GpIA7 lines compared with the control roots (see Supplementary Fig. S4). The unchanged infection rate suggests that additional GpIA7 does not enhance already successful interactions.

### StEBP1 is a putative host target of GpIA7 in potato

A Y2H approach was used to identify potential interacting protein partner(s) of GpIA7 in potato. A prey library was made from *G. pallida*-infected potato roots (see the Materials and methods). Screening by mating of the prey library with the GpIA7 bait generated >15 million diploids, of which at least 400 grew under high stringency selection and activated all reporters. Direct sequencing from diploids of 55 PCR-amplified prey clone inserts identified 23 independent clones. The most abundant prey clone, identified in 15 of the 55 diploids investigated, was a 533 bp fragment of the potato *EBP1* gene, corresponding to the C-terminal end of the protein and the 3′-untranslated region sequence.

**Fig. 3.**
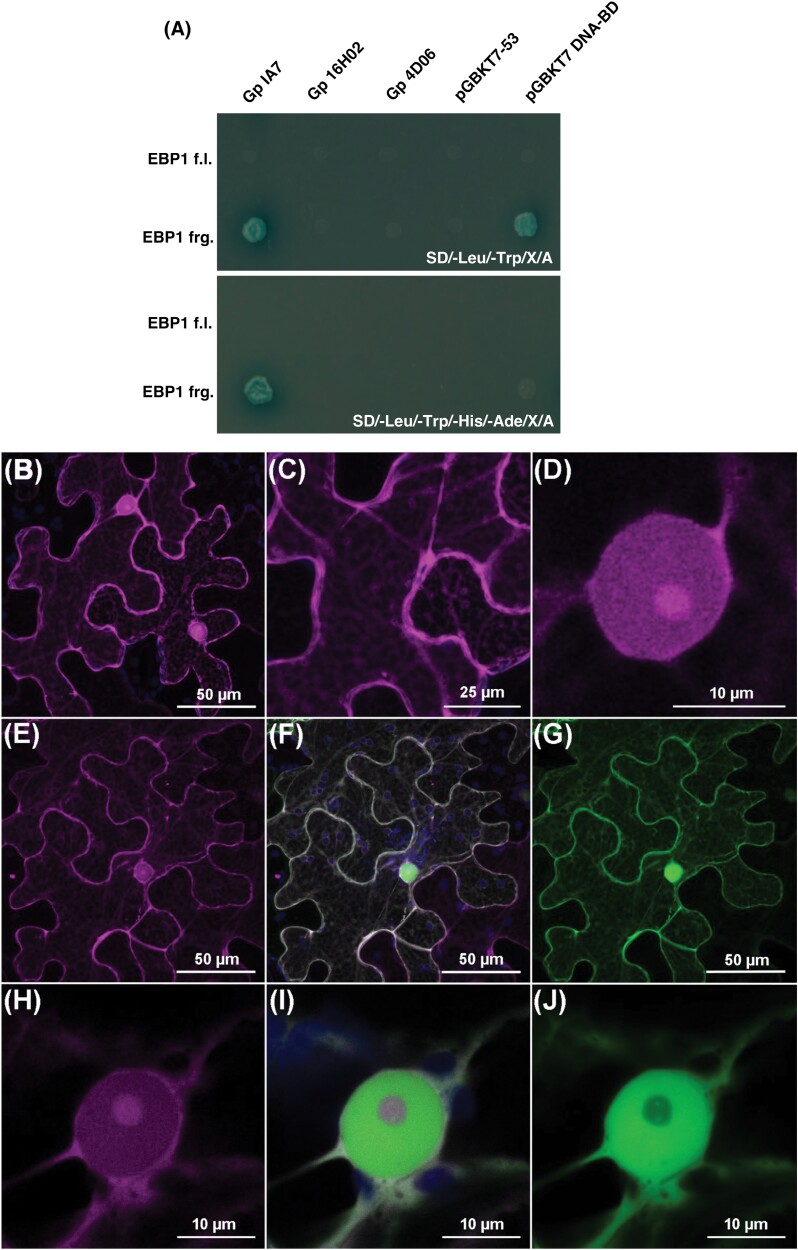
GpIA7 and the potato EBP1 protein interact in yeast and co-localize *in planta*. (A) The StEBP1 fragment (EBP1 frg) identified during yeast two-hybrid screening interacts with the GpIA7 effector on both low- (top panel selection) and high- (bottom panel selection) stringency media. Specificity was confirmed by the failure of the StEBP1 protein fragment to interact on high-stringency media with either the murine p53 protein (pGBKT7-53), two unrelated effectors from *G. pallida* (Gp4D06 and Gp16H02), or the BD domain from the empty pGBKT7 DNA-BD cloning vector. Full-length StEBP1 (EBP1 f.l) did not interact with any of the bait proteins tested. (B–J) Subcellular localization of StEBP1 and co-localization with GpIA7 *in planta. Agrobacterium*-mediated transient expression of *StEBP1* tagged with mRFP as an *mRFP::StEBP1* fusion in *Nicotiana benthamiana* leaves, either alone (B–D) or in combination with the *eGFP::GpIA7* gene fusion [(E, H), red channel; (G, J), green channel; (F, I) overlay]. Close-up images of the nuclei (H–J). Pictures were taken 2 d post-infiltration by confocal microscopy. Images were collected as *z*-stacks, and the median section through the nucleolus is presented, with mRFP displayed in magenta, eGFP in green, and autofluorescence from chloroplasts in blue. Each localization experiment was replicated at least twice.

The *StEBP1* clone was rescued from one of the yeast diploids and the interaction with GpIA7 re-tested by mating of separate strains. The full-length *StEBP1* coding sequence (see Supplementary Fig. S1A) was cloned from potato cv. Désirée and tested alongside the original truncated fragment in Y2H. GpIA7 reproducibly interacted with the StEBP1 fragment but not with the full-length protein (Fig. 3A). The murine p53 protein and two unrelated *G. pallida* effectors (Gp4D06 and Gp16H02) were used as control bait proteins to test the specificity of the interaction (Fig. 3A), and showed no interaction with either the truncated or the full-length StEBP1 protein (Fig. 3A). This suggests that GpIA7 physically interacts with a region within the last 109 amino acids of the StEBP1 protein (Supplementary Fig. S1B), in a specific and reproducible manner. The conformation adopted by full-length StEBP1 in yeast may have either prevented physical interaction with GpIA7 or impaired activation of the reporters (Clontech Matchmaker Gold Yeast Two-Hybrid System User Manual; Lambert *et al.*, 2016). StEBP1 is a regulator of plant growth that controls organ size in a dose- and auxin-dependent manner and influences the timing of vegetative to reproductive transition (Horváth *et al*., 2006; Cheng *et al.*, 2016). It is present in all growing tissues including roots (Horváth *et al*., 2006). Furthermore, *AtEBP1* (At3g51800) transcripts have been reported within syncytia induced by a cyst nematode in roots of *Arabidopsis thaliana* (Szakasits *et al.*, 2009). Taken together, these characteristics endorsed StEBP1 as a candidate for further analysis.

### GpIA7 and StEBP1 interact in plant cells

The subcellular localization of StEBP1 *in planta* was analysed, to determine if it may be present in the same cellular compartments as GpIA7. N-terminally tagged full-length StEBP1 protein (mRFP::StEBP1) transiently expressed in leaf epidermal cells of *N. benthamiana* localized in the cytoplasm and the nucleus, where it strongly accumulated in the nucleolus (Fig. 3B–D; see Supplementary Fig. S5G–I). This pattern is consistent with the nuclear, nucleolar, and cytoplasmic localization reported for characterized EBP1 proteins in other organisms (Karlsson *et al.*, 2016; Li *et al.*, 2018; Lokdarshi *et al.*, 2020). The subcellular localization of StEBP1 remained visibly unchanged in the presence of the eGFP-tagged effector (eGFP::GpIA7; Fig. 3E, F, H, I). GpIA7 and its putative target, StEBP1, perfectly co-localize in both the cytoplasm and nucleoplasm (Fig. 3F, G, I, J; Supplementary Fig. S4A–L). However, there was no obvious accumulation of the effector in the nucleolus (Fig. 3I, J).

**Fig. 4.**
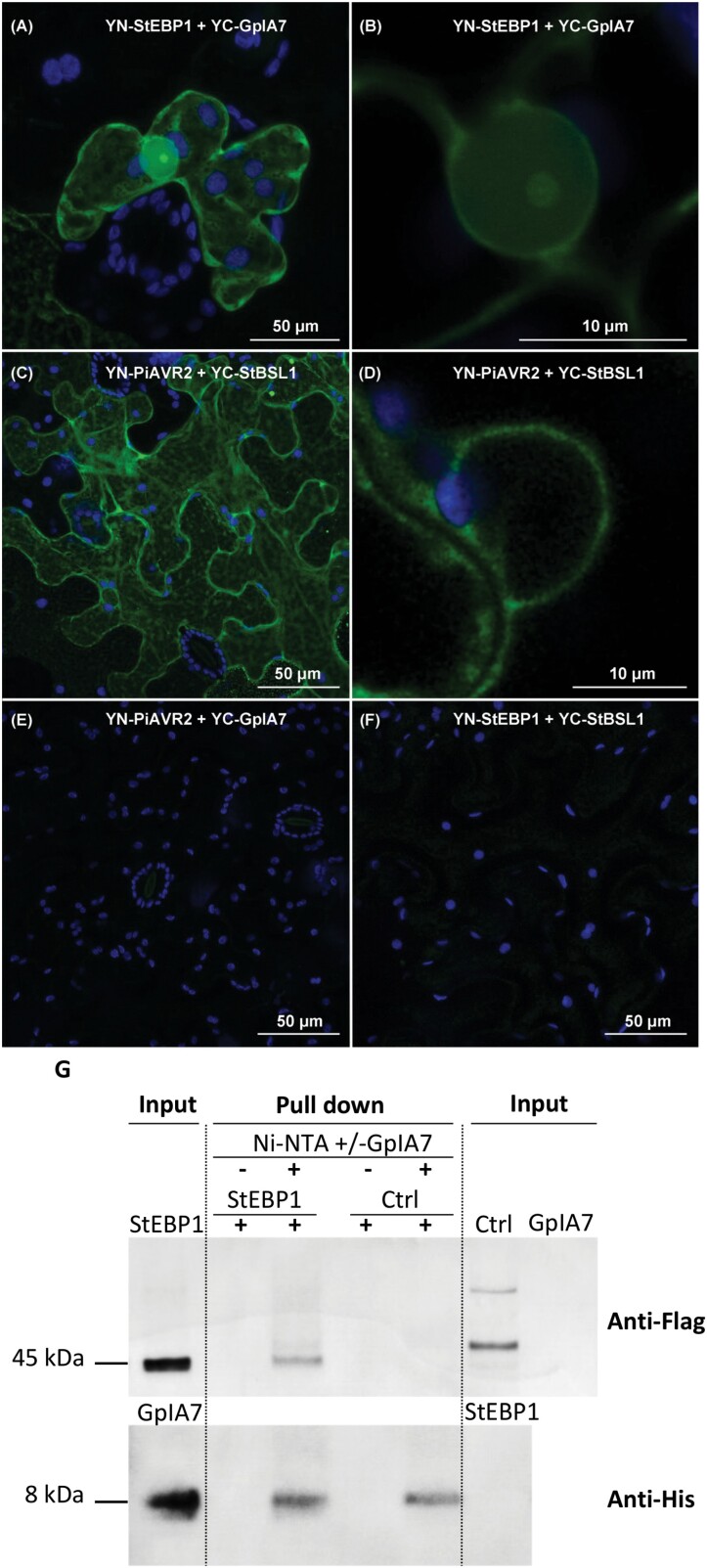
GpIA7 specifically associates with StEBP1 in BiFC and pull-down assays. (A–F) BiFC assay using transient expression in *N. benthamiana* of split-yellow fluorescent protein (split-YFP) fusion pairs indicated in subpanels. Pictures were taken 2 d post-infiltration by confocal microscopy. Images were collected as *z*-stacks, (A, C, E, F) and presented as maximum projection, and (B, D) are median section through the nucleolus for a close-up picture of the nucleus from the same cell. Reconstituted YFP is displayed in green, and autofluorescence from chloroplasts in blue. The experiment was replicated with a similar pattern. (G) Ni-NTA-immobilized GpIA7 pulls down StEBP1 from a plant cell lysate. Western blots of input and eluted proteins were probed with anti-Flag and anti-His antibodies. 3×FLAG-StEBP1 was pulled down from plant cell lysate by the Ni-NTA-immobilized 6×His-GpIA7 (Ni-NTA+GpIA7) but not by the Ni-NTA resin alone (Ni-NTA–GpIA7). The control protein, 3×FLAG-GS22, was not pulled down in either case. The bacterial cell lysate and plant cell lysate were tested for cross-reactivity. The experiment was repeated in triplicate, and a representative result is shown.

A BiFC assay was used to observe where the interaction of GpIA7 and StEBP1 may take place *in planta*. Both proteins were transiently co-expressed in the leaves of *N. benthamiana* using split-YFP fusions. The YFP fluorescence was observed in the nucleolus, nucleoplasm, and cytoplasm, indicating that YC–GpIA7 and YN–StEBP1 fusion proteins were in close proximity in these different plant cellular compartments, allowing reconstitution of the YFP to occur (Fig. 4A, B). The fluorescence signal was apparently as strong as the one resulting from the interaction between the control protein pair YC–StBSL1 and YN–PiAVR2 (Fig. 4C, D), which was in that case restricted to the cytoplasm in agreement with the original characterization of these proteins (Saunders *et al.*, 2012). When either YC–GpIA7 or YN–StEBP1 was expressed in combination with the complementary YC control proteins, only faint background YFP signal was observed (Fig. 4E, F), confirming that the protein complex formed between YC–GpIA7 and YN–StEBP1 was genuine.

Although the BiFC assay indicated that the interaction between GpIA7 and StEBP1 could occur in the nucleolus (Fig. 4A, B), the localization study did not provide strong evidence for an accumulation or re-localization of the effector in the nucleolus (Figs 1C, 3E). We therefore quantified the eGFP signal of the eGFP::GpIA7 fusion protein alone or in the presence of the mRFP::StEBP1 fusion in the nucleoplasm and nucleolus, compared with cells that transiently expressed free eGFP only. The free eGFP had a nucleolus/nucleoplasm signal ratio of 0.5, indicating that the protein was present in both the nucleoplasm and nucleolus, but was more abundant in the former. The ratio for the eGFP::GpIA7 fusion proved to be significantly higher, indicating that indeed GpIA7 slightly accumulates in the nucleolus, a localization that was observed irrespective of the presence of StEBP1 (see Supplementary Fig. S4M). Altogether, the BiFC and localization studies suggest that GpIA7 and its StEBP1 target may interact in either subcellular compartment in which they are present.

An *in vitro* pull-down assay was performed to provide confirmation of a physical interaction. Ni-NTA-immobilized GpIA7 expressed as a 6×His-tag fusion was used to pull down StEBP1 protein fused with the 3×FLAG tag from a plant cell lysate. Western blot analysis revealed that StEBP1 did bind to Ni-NTA-immobilized GpIA7 but not to Ni-NTA alone (Fig. 4G). An unrelated 3×FLAG-tagged protein, GpGS22 (Lilley *et al.*, 2018), was similarly expressed in *N. benthamiana* leaves and used as a negative control to discount binding of the 3×FLAG tag to GpIA7. This protein did not bind either to immobilized GpIA7 or to Ni-NTA alone (Fig. 4G).

### Silencing *StEBP1* does not alter susceptibility of potato to *G. pallida*

To evaluate the impact of *StEBP1* on potato susceptibility to *G. pallida*, transgenic EBP1 (RNAi) lines were generated for the susceptible cv. Désirée. Silencing of *StEBP1* had a large impact on the plant phenotype. Most of the regenerated lines were smaller than control plants and showed retarded growth with poor rooting, which was consistent with previous studies (Horváth *et al*., 2006). The most severely affected line, EBP1-9 (RNAi) with greatest reduction in *StEBP1* transcript, did not survive transfer to glasshouse conditions. Therefore, two lines, EBP1-5 (RNAi) and EBP1-7 (RNAi), with medium to normal phenotype in which the level of silencing correlated with the severity of the phenotype, were chosen for these experiments (see Supplementary Figs S6, S7).

**Fig. 5.**
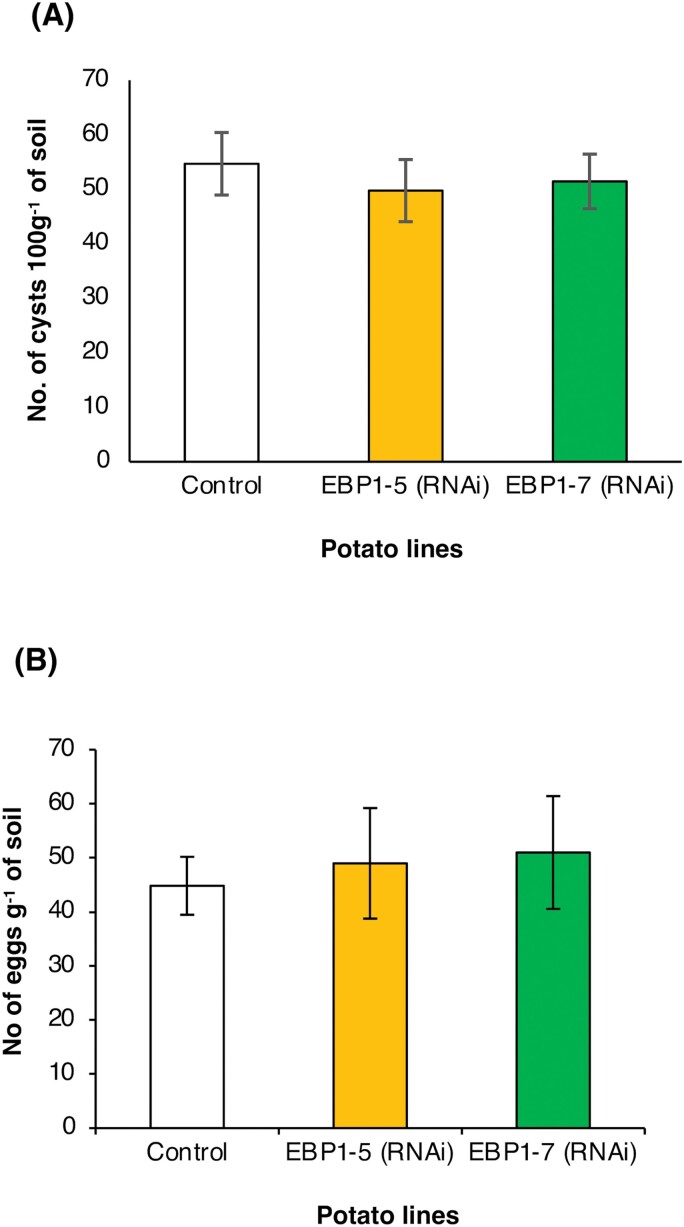
Susceptibility to *Globodera pallida* infection is not affected by *StEBP1* silencing in potato. Plants from the transgenic StEBP1 (RNAi) lines or mock-transformed control plants were grown in soil infected with 30 cysts of *G. pallida* per pot. The nematode infection measured as cysts (A) and eggs g^−1^ of soil (B) was determined at senescence of the plants. There were no significant differences observed between control and silenced plants in the number of cysts 100 g^−1^ of soil or eggs g^−1^ soil retrieved at the end of the trial. Values are means, and the error bars are the SE (*n*=11, 10, and 9, respectively; ANOVA).

The EBP1 (RNAi) lines were planted in *G. pallida*-infested soil, and at natural senescence the number of cysts and eggs g^−1^ of soil was determined. Neither the infectivity of the nematode nor the reproduction of *G. pallida* was affected by the reduction of *StEBP1* transcripts in the EBP1 (RNAi) lines compared with the control (Fig. 5A,B). Therefore, *StEBP1* silencing had no significant impact on the plant susceptibility to *G. pallida.* The EBP1-5 (RNAi) line did have significantly less root mass than the control plants (see Supplementary Fig. S8) whilst root mass of EBP1-7 (RNAi) was similar to that of the control (Supplementary Fig. S8).

### The expression of potato cell cycle genes *StCYCD3;1, StRBR1,* and *StRNR2* is reduced in *GpIA7* overexpression lines

StEBP1 promotes cell proliferation in meristems, while in post-mitotic cells it enhances cell expansion through modulation of key cell cycle components. StEBP1 positively regulates the expression of the cell cycle genes *StCYCD3;1*, *StRNR2*, and *StCDKB1;1*, with reduced expression of these genes in StEBP-1 (RNAi) lines (Horváth *et al*., 2006).

**Fig. 6.**
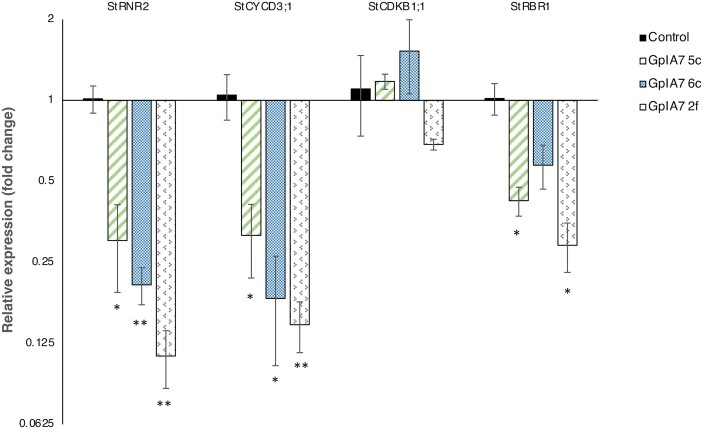
Relative expression of cell cycle genes in *GpIA7* overexpression potato lines. Expression of *StCYCD3;1*, *StRNR2*, *StCDKB1;1*, and *StRBR1* was determined by qRT–PCR in the leaves of three independent GpIA7 lines (5c, 6c, and 2f) and in the mock-transformed control line. The fold changes in expression were calculated as a ratio between the relative expression (using *StEF1α* for normalization) of the GpIA7 lines and the control line. Values are mean fold changes, and error bars represent the SE. At least three biological replicates and two technical replicates were used; *P<0.05, **P<0.01) (ANOVA, followed by Bonferroni post-hoc correction).

To explore if GpIA7 interferes with the function of StEBP1, we quantified the expression of *StCYCD3;1*, *StRNR2*, and *StCDKB1;1* in the young leaves, of the same developmental stage, of three different potato lines overexpressing *GpIA7*. The expression level of two genes, *StCYCD3;1* and *StRNR2*, was reduced between 69% and 85%, and 70% and 88%, respectively, in all transgenic lines tested when compared with control plants (Fig. 6). There was no significant change, however, in the abundance of *StCDKB1;1* transcripts (Fig. 6). We hypothesize that GpIA7 may therefore alter the transcriptional regulation of key cell cycle genes through its association with StEBP1.

*StRBR1* is a major regulator gene of the cell cycle, and the level of StRBR1 protein is known to be modulated by StEBP1 (Horváth *et al*., 2006). Its expression was also significantly reduced by 58–71% in two out of the three GpIA7 overexpression lines tested (Fig. 6). To confirm that this reduction was most probably mediated through StEBP1, we determined the level of *StRBR1* transcripts in the leaves of three StEBP1 (RNAi) lines. The results indicate that *StRBR1* is similarly down-regulated in these lines that have reduced levels of *StEBP1* expression (see Supplementary Fig. S9) so establishing the basis for a regulatory connection between GpIA7, StEBP1, and StRBR1.

## Discussion

The potato cyst nematode *G. pallida* generates a new structure, the syncytium, in the differentiated zone of the host root, reactivating the cell cycle and differentiation processes. The normal cell cycle programme is modified, with the nuclei undergoing several rounds of endoreduplication (de Almeida Engler *et al.*, 1999), leading to increased ploidy. However, the molecular mechanism and the nematode effectors involved in initiating endoreduplication and suppressing mitosis remain to be identified (de Almeida Engler and Gheysen, 2013; Vieira *et al.*, 2013). In this study, we provide an insight into how the potato cyst nematode may influence the host cell cycle and induce endocycling. Our data suggest that the *G. pallida* GpIA7 effector reduces the expression of key cell cycle regulators, through association with StEBP1 in host plants, thereby stimulating the endocycle when syncytium formation is triggered. An orthologue of *GpIA7* is reported in *Globodera mexicana* (Blanchard *et al.*, 2007), and similar sequences can be identified in the draft genomes of *G. rostochiensis* and *H. glycines*, suggesting a common mechanism of syncytium initiation. Consistent with such a role in the early stages of parasitism, the GpIA7 effector is expressed maximally at the pre- and post-parasitic J2 stages and is secreted from the subventral glands of *G. pallida* that are most active at this time (Lilley *et al.*, 2005).

The onset of endoreduplication in plants is determined by a controlled reduction of mitotic CDK activity (Breuer *et al.*, 2014). This can be achieved by several mechanisms including selective degradation of cyclins (Kondorosi and Kondorosi, 2004; De Veylder *et al.*, 2011; Takahashi and Umeda, 2014), interaction with CDK inhibitors (Churchman *et al.*, 2006), and transcriptional regulation of cyclin gene expression (Joubès *et al.*, 1999; Sun *et al.*, 1999). Our results suggest that the third of these mechanisms is modulated by a cyst nematode effector.

The transcript level of *CYCD3;1*, an intriguing type D cyclin that promotes mitosis and supresses the endocycle (Schnittger *et al.*, 2002; Dewitte *et al.*, 2003; Menges *et al.*, 2006), was reduced in *GpIA7*-overexpressing potato lines (Fig. 6). It has been suggested that CYCD3;1 might act as a mitotic cyclin as, unlike other genes operating at the G_1_/S boundary that are generally associated with S phase entry, it promotes a specific type of cycle (Dewitte *et al.*, 2007). This idea is supported by recent findings that showed that, at least in developing trichomes, CYCD3;1 can act independently of CYC/CDKB1 complexes to promote mitosis, implying its uniqueness among D cyclins (Wang *et al.*, 2020). It has been hypothesized that during the mitotic cycle the CYCD3/CDKA;1 complexes might have a special role in phosphorylating G_2_/M transcription factors, such as MYB3Rs (Wang *et al.*, 2020). Furthermore, *CYCD3;1* transcripts are expressed at their highest level in G_2_/M, rather than S-phase (Menges *et al.*, 2005), a possible consequence of *CYCD3;1* genes being directly regulated through the binding of E2FB to their promoter (Őszi *et al.*, 2020). A reduction of CYCD3 levels characterizes the transition from mitotic cell cycle to endocycle (Dewitte *et al.*, 2007); therefore, such a reduction might be expected in the developing syncytium if its nuclei are subjected to several rounds of endoreduplication. In Arabidopsis, high levels of *CYCD3;1* strongly inhibit the endocycle, promoting cell proliferation (Schnittger *et al.*, 2002; Dewitte *et al.*, 2003; Menges *et al.*, 2006). The CYCD3/CDKA;1 complexes were found to be the target of SIM CDK inhibitors, which are key factors in the mitotic to endocycle transition in developing trichomes (Churchman *et al.*, 2006; Kumar *et al.*, 2015). The loss of *CYCD3* activity triggers premature onset of the endocycle in leaves and initiation of the endocycle in tissues that do not normally endoreduplicate (Dewitte *et al.*, 2007). Interestingly, *AtCYCD3;1* loss of function also triggers late onset of flowering, which is a phenotype we observed in two of three potato lines overexpressing *GpIA7* (Fig. 2C).

The overexpression of *GpIA7* in potato plants also reduced *StRBR1* transcripts in leaves (Fig.6). RBR1 is a master regulator of the cell cycle and probably functions as a molecular hub integrating different signals to fine-tune the cell cycle activity (Desvoyes *et al.*, 2014; Harashima and Sugimoto, 2016). As a single-copy gene in dicotyledonous plants, *RBR1* is particularly sensitive to dosage imbalance (Desvoyes *et al.*, 2014). It is a suppressor of the conserved E2F family of transcription factors. Upon phosphorylation by CDKA;1 in complex with CYCD3;1 (Dewitte *et al.*, 2003; Menges *et al.*, 2006), RBR1 dissociates from E2FB, which then activates the expression of downstream S-phase-related genes to regulate the G_1_/S transition. Similarly, RBR1 forms a stable repressor complex with E2FA to inhibit the expression of a set of genes involved in the endocycle (Magyar *et al.*, 2012). It was suggested that RBR1 in association with E2FA plays a role in maintaining proliferation competence in the meristem (Magyar *et al.*, 2012; Őszi *et al.*, 2020). In plants, RBR1 is abundant in meristematic cells, but its level decreases as development proceeds (Borghi *et al.*, 2010; Magyar *et al.*, 2012). Thus, silencing *RBR1* stimulates endoreduplication in post-mitotic cells (Park *et al.*, 2005; Desvoyes *et al.*, 2006; Jordan *et al.*, 2007), while conversely an elevated level of RBR1 represses endoreduplication (Magyar *et al.*, 2012). The reduction in both *StCYCD3;1* and *StRBR1* transcripts, as a consequence of *GpIA7* overexpression, is therefore consistent with a potential role for the GpIA7 effector in triggering the onset of endoreduplication in early-stage syncytia. The down-regulation of these cell cycle genes could also explain the phenotypic changes in growth and development observed in the *GpIA7*-overexpressing lines.

The reduced expression of these key cell cycle regulators most probably occurs through the association of GpIA7 with StEBP1. Our Y2H, BiFC, and pull-down results suggest that the StEBP1 protein is a host target for the GpIA7 effector (Figs 3A, 4). *GpIA7*-overexpressing potato lines (Fig. 2A, B, D) displayed a milder form of the phenotype of the StEBP1 (RNAi) lines (Horváth *et al*., 2006), indicating that GpIA7 hinders rather than stimulates StEBP1 activity. The down-regulation of several of the downstream EBP1 targets—*CYCD3;1*, *RBR1*, and *RNR2*—in *GpIA7*-overexpressing lines (Fig. 6) further supports this theory. Although StEBP1 has been reported as a negative regulator of StRBR1 protein (Horváth *et al*., 2006), the effect on the *StRBR1* transcript was unknown. We found that, consistent with our hypothesis, *StRBR1* was actually down-regulated in StEBP1 (RNAi) lines (Supplementary Fig. S9), implicating StEBP1 as a positive regulator of *StRBR1* expression.

The cell cycle progression is conditioned by particular thresholds in the activity of CDKs and cyclins (De Veylder *et al.*, 2007), and as such it is impossible to accurately predict if the cell cycle components targeted by the GpIA7 effector reach these thresholds. The effects of *GpIA7* overexpression in plants cannot be directly correlated with the situation in syncytia as the concentration of the effector in the feeding cell is not known. Also, the onset and progression of endoreduplication is likely to be the outcome of multiple effectors acting in a coordinated manner. The action of a single effector can only provide a fragmented understanding of the process. Detailed transcriptomic analysis of syncytia induced by *G. pallida* in potato over the first few days of parasitism may provide insights, although the small size of the developing syncytia at this early stage would make this technically challenging. Although most studies focus on leaves, *EBP1* is expressed in root tissue as well, predominantly in meristematic cells, but also at a lower level in the elongation and differentiation zone (Lokdarshi *et al.*, 2020), providing further support for a role in syncytium formation. Despite displaying phenotypic changes in growth and development, the *GpIA7*-overexpressing plants did not manifest altered susceptibility to *G. pallida*, suggesting that invasion and establishment of the nematodes were not affected (Supplementary Fig. S4). Ectopic expression of effectors in host plants is often reported to increase susceptibility to the nematode (Lee *et al.*, 2011; Hewezi *et al.*, 2015); however, this is not always the case. It has been suggested that the level of an effector introduced into the syncytium by the nematode is already sufficient for successful parasitism, such that additional effector protein supplied by a transgenic host does not always provide an advantage (Barnes *et al.*, 2018). GpIA7 contributes to the down-regulation of cell cycle components, and it is possible that above a particular threshold there is no further benefit to the nematode. Indeed, in a tightly controlled complex system, excess of a single effector, when multiple effectors are likely to be required to achieve an optimal outcome, may even be detrimental.

Modulation of ploidy level at, or adjacent to, the site of infection is a common feature for many plant pathogens (Wildermuth, 2010), with components of the plant cell cycle regulatory system known to be affected. Plant DNA synthesis and the cell cycle are activated in differentiated leaf cells of maize by the biotroph *Ustilago maydis* during the infection process (Redkar *et al.*, 2017). The expression of *CYCD3;2* is reduced by the *Cabbage leaf curl virus* in host plants; however, the effector impacting on the host target was not identified (Ascencio-Ibanez *et al*., 2008). RBR1 is also a target for several viral effectors (Aronson *et al.*, 2000; Gutierrez, 2000; Arguello-Astorga *et al.*, 2004) that do not alter the expression level but bind to RBR1 in the host plants, thus releasing the E2F factors from repression by RBR1 (Desvoyes *et al.*, 2006).

In summary, we show that the GpIA7 effector from *G. pallida* targets the cell growth regulator StEBP1 and hinders its normal functioning in the host. The expression levels of several StEBP1 downstream targets, which are major regulators of the cell cycle that are also involved in the onset of the endocycle, are altered as a result of the activity of GpIA7 in the plant cell. Such a perturbation to a finely regulated system could play a role in triggering the endoreduplication that characterizes nematode-induced syncytia in host roots. Further research will be necessary to address the impact of *StRBR1* and *StCycD3* down-regulation on other core cell cycle genes and whether interaction with GpIA7 can also affect regulation of translational processes by StEBP1.

## Supplementary data

The following supplementary data are available at *JXB* online.

Fig. S1. The sequences of EBP1 and GpIA7 used in this study; spatial and temporal analysis of GpIA7 transcripts.

Fig. S2. Validation of the use of EF1-α as the reference gene in this study.

Fig. S3. Evaluation by qRT–PCR of *GpIA7* expression in *Globodera pallida* J2s treated with GpIA7 dsRNA.

Fig. S4. Evaluation of the susceptibility of GpIA7 transgenic overexpression potato lines to *Globodera pallida.*

Fig. S5. Subcellular localization of GpIA7 and StEBP1 *in planta.*

Fig. S6. The down-regulation of *StEBP1* through RNAi significantly alters the growth of the transgenic plants.

Fig. S7. Evaluation of *StEBP1* gene silencing in the EBP1 (RNAi) potato lines.

Fig. S8. Evaluation of the root mass of EBP1 (RNAi) potato lines.

Fig. S9. Relative expression of the *StRBR1* gene in EBP1 (RNAi) potato lines.

Table S1. Primers used in this study

erab353_suppl_Supplementary_Figures_S1-S9_Table_S1

## Data Availability

All data supporting the findings of this study are available within the paper and within its supplementary data published online. Any other related information is available upon request from the corresponding author (Peter Urwin).
