# Supplementary material for: The GpIA7 effector from the potato cyst nematode Globodera pallida targets potato EBP1 and interferes with the plant cell cycle
Source: J Exp Bot. 2021 Jul 26;72(20):7301–15. doi: 10.1093/jxb/erab353 (PMC8547150; doi:10.1093/jxb/erab353)
Supplement: erab353_suppl_Supplementary_Figures_S1-S9_Table_S1 [file erab353_suppl_supplementary_figures_s1-s9_table_s1.pdf]

Supplementary Data

Article title: The GpIA7 effector from the potato cyst nematode *Globodera pallida* targets potato EBP1 and interferes with the plant cell cycle programme

Authors: Mirela C. Coke[1], Sophie Mantelin[2,a], Peter Thorpe[1,2,b], Catherine J. Lilley[1], Kathryn M. Wright[2], Daniel S. Shaw[1], Adams Chande[1], John T. Jones[2,3], Peter E. Urwin[1*]

The following Supplementary data is available for this article:

Fig. S1 The sequences of StEBP1 and GpIA7 used in this study; spatial and temporal analysis of GpIA7 transcripts.

Fig. S2 Validation of *StEF1-α* as reference gene for qRT-PCR analysis in this study.

Fig. S3 Evaluation by qRT-PCR of *GpIA7* expression in *Globodera pallida* J2s treated with GpIA7 dsRNA.

Fig. S4 Evaluation of the susceptibility of GpIA7 transgenic overexpression potato lines to *Globodera pallida.*

Fig. S5 The assessment of the nucleolus:nucleoplasm partitioning of GpIA7 in plant cell nuclei.

Fig. S6 Downregulation of *StEBP1* through RNAi significantly alters the growth of the transgenic plants.

Fig. S7 Evaluation of *StEBP1* gene silencing in the EBP1 *(RNAi*) potato lines.

Fig. S8 Evaluation of the root mass of EBP1 *(RNAi)* potato lines.

Fig. S9 Relative expression of the *StRBR1* gene in EBP1 *(RNAi)* potato lines.

Table S1 Primers used in this study

(A)

StEBP1

```
   1 ATGTCGGACG ACGAGAGAGA AGAGAAAGAA TTGGATCTCA CAAGTCCTGA GGTCGTCACC
  61 AAGTACAAGA GCGCCGCTGA AATTGTTAAC AAGGCGCTGC AGTTGGTGTT GTCCGAATGC
 121 AAGCCAAAAG CAAAGATAGT TGATCTTTGT GAGAAAGGGG ATGCCTTTAT CAAAGAGCAA
 181 ACTGGAAATA TGTACAAGAA TGTGAAGAAG AAGATTGAGA GAGGTGTTGC ATTTCCAACA
 241 TGTATTTCAG TTAATAACAC CGTGTGCCAT TTCTCTCCAT TGGCTAGTGA TGAGACAGTA
 301 GTGGAAGAAG GTGATATATT GAAGATTGAT ATGGGATGTC ACATTGATGG ATTTATTGCA
 361 GTAGTTGGAC ATACACATGT TCTTCACGAA GGACCAGTTA CTGGTAGAGC TGCTGATGTC
 421 ATTGCAGCTG CTAATACAGC TGCTGAAGTT GCTTTGAGAC TTGTAAGACC AGGAAAGAAG
 481 AACTCGGATG TAACAGAAGC TATTCAGAAG GTTGCTGCTG CCTATGACTG CAAGATTGTC
 541 GAGGGTGTAT TGAGCCATCA AATGAAGCAG TTTGTTATTG ATGGAAACAA AGTTGTATTG
 601 AGCGTGTCCA ATCCTGACAC AAGAGTAGAT GAGGCAGAAT TTGAAGAGAA TGAGGTCTAC
 661 TCCATTGATA TCGTGACGAG CACTGGTGAT GGAAAGCCCA AGTTGTTGGA TGAGAAACAA
 721 ACAACCATCT ACAAGAGAGC TGTGGACAAA AGCTATAACC TGAAGATGAA AGCCTCAAGG
 781 TTCATCTTCA GTGAAATCAG TCAGAAGTTC CCTATCATGC CATTTACCGC AAGGGATTTG
 841 GAGGAGAAGA GGGCTCGTTT GGGCCTTGTT GAATGTGTTA ACCATGAGCT TTTGCAGCCA
 901 TATCCTGTTC TACATGAGAA ACCTGGTGAT TTGGTTGCTC ACATTAAGTT CACAGTGCTG
 961 TTAATGCCCA ATGGATCGGA CAGGGTAACA TCTCATGCGC TCCAGGAGCT TCAGCCTACA
1021 AAGACAACAG AGAATGAACC TGAAATCAAG GCTTGGCTAG CCCTTCCCAC CAAGACTAAG
1081 AAGAAAGGTG GTGGGAAGAA AAAGAAAGGA AAGAAAGGTG ACAAGGTAGA AGAGGCATCT
1141 CAAGCTGAGC CTATGGAAGG ATAG
```

(B)

```
StEBP1
  1 MSDDEREEKE LDLTSPEVVT KYKSAAEIVN KALQLVLSEC KPKAKIVDLC EKGDAFIKEQ
 61 TGNMYKNVKK KIERGVAFPT CISVNNTVCH FSPLASDETV VEEGDILKID MGCHIDGFIA
121 VVGHTHVLHE GPVTGRAADV IAAANTAAEV ALRLVRPGKK NSDVTEAIQK VAAAYDCKIV
181 EGVLSHQMKQ FVIDGNKVVL SVSNPDTRVD EAEFEENEVY SIDIVTSTGD GKPKLLDEKQ
241 TTIYKRAVDK SYNLKMKASR FIFSEISQKF PIMPFTARDL EEKRARLGLV ECVNHELLQP
301 YPVLHEKPGD LVAHIKFTVL LMPNGSDRVT SHALQELQPT KTTENEPEIK AWLALPTKTK
361 KKGGGKKKKG KKGDKVEEAS QAEPMEG
```

(C)

```
GPLIN_000740500      ATGAATTTTCAAATTTTCTTTTATCAAACGGTGTGCGTTTTGTTGCTGATTTCAACGACA
GPLIN_000638300      ATGAATTTTCAAATTTTCTTTTATCAAACGGTGTGCGTTTTGTTGCTGATTTCAACGACA
GpIA7                ATGAATTTTCAAATTTTCTTTTATCAAACGGTGTGCGTTTTGTTGCTGATTTCAACGACA
                     ************************************************************

GPLIN_000740500      GACTTTGTGGCTTCGCAGGACGCTGCTCCCATCACCAAGGCGTCGTCCTCAAGCTGTACC
GPLIN_000638300      GACTTTGTGGCTTCGCAGGACGCTGCTCCCATCACCAAGGCGTCGTCCTCAAGCTGTACC
GpIA7                GACTTTGTGGCTTCGCAGGACGCTGCTCCCATCACCAAGGCGTCGTCCTCAAGCTGTACC
                     ************************************************************

GPLIN_000740500      GACCCGGCTGGCACCGATCAGTGCAATTATTACAAAAGGTACTGCAACCAATACAAGGGA
GPLIN_000638300      GACCCGGCTGGCCCCGATCAGTGCAATTATTACAAAAGGTACTGCAACCAATACAAGGGA
GpIA7                GACCCGGCTGGCACCGATCAGTGCAATTATTACAAAAGGTACTGCAACCAATACAAGGGA
                     ************ ***********************************************

GPLIN_000740500      ATGCTGACAGCCATGTGCCCAAAAACCTGCAAGTTTTGC
GPLIN_000638300      ATGCTGAAAACGATGTGCCCAAAAACTTGCAAGTTTTGC
GpIA7                ATGCTGAAAACGATGTGCCCAAAAACCTGCAAGTTTTGC
                     ******* * * ************** ************
```

(D)

```
GPLIN_000740500      MNFQIFFYQTVCVLLLISTTDFVASQDAAPITKASSSSCTDPAGTDQCNYYKRYCNQYKG
GPLIN_000638300      MNFQIFFYQTVCVLLLISTTDFVASQDAAPITKASSSSCTDPAGPDQCNYYKRYCNQYKG
GpIA7                MNFQIFFYQTVCVLLLISTTDFVASQDAAPITKASSSSCTDPAGTDQCNYYKRYCNQYKG
                     ******************************************** ***************
```

```
GPLIN_000740500        MLTAMCPKTCKFC
GPLIN_000638300        MLKTMCPKTCKFC
GpIA7                  MLKTMCPKTCKFC
                       **.:*********
```

(E)

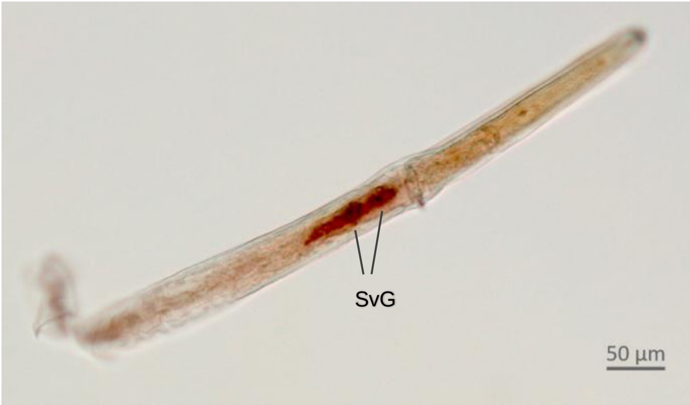

(F)

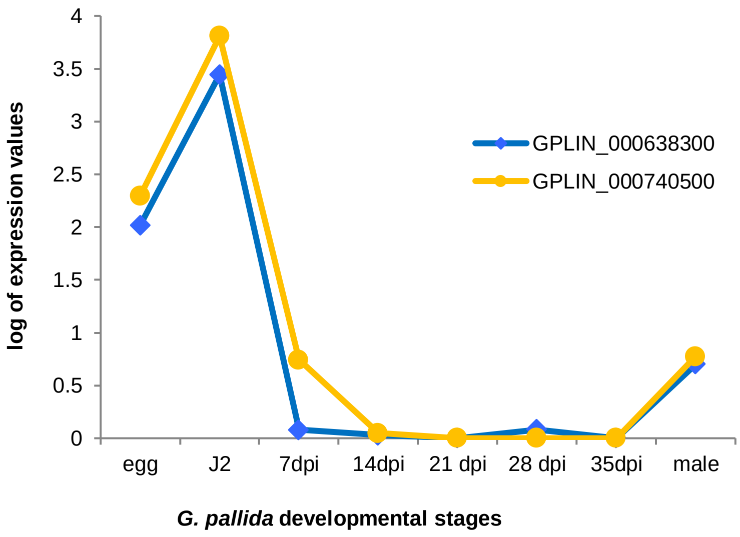

(G)

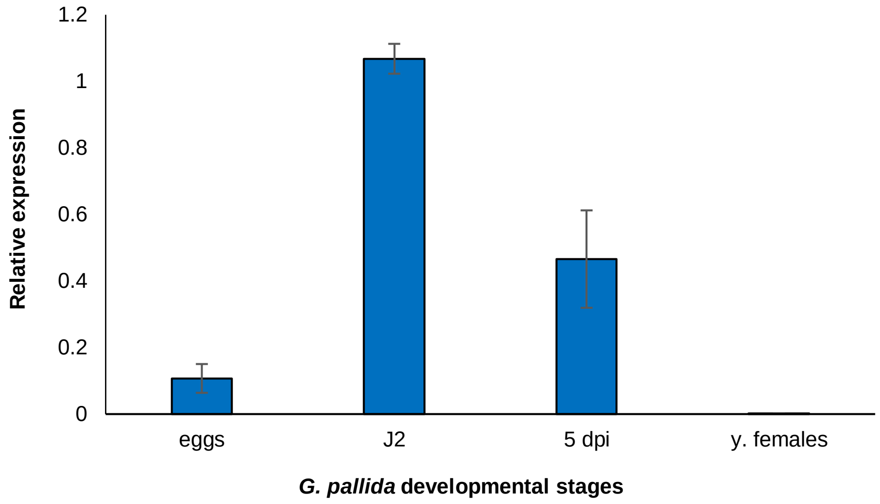

Fig. S1 The sequences of StEBP1 and GpIA7 used in this study; spatial and temporal analysis of GpIA7 transcripts. (A,B) The complete *StEBP1* nucleotide (A) and amino acid (B) sequence isolated in this study. Green highlighted nucleotides/ amino acids correspond to the fragment identified during the Y2H screen (C,D) Nucleotide (C) and amino acid (D) sequence comparison of GPLIN_000638300, GPLIN_000740500 and the *GpIA7* sequence isolated in this study. Red boxes indicate the signal peptide while yellow highlighted nucleotides/amino acids correspond to the ShK domain. Identical bases/amino acids, conservative substitutions, and semi-conservative substitutions are indicated by (∗), (:), and (.), respectively. (E) Localisation of *GpIA7* transcripts. The expression of *GpIA7* was localised in the subventral glands (SvG) of *Globodera pallida* pre-parasitic J2 by *in situ* hybridization as described by Thorpe et al. (2014). The probe used detects both *GpIA7* paralogues. (F) Relative expression of *GpIA7* (GPLIN_000638300) and its paralogue (GPLIN_000740500) during the life cycle of *G. pallida*. The expression profiles of two *GpIA7* paralogues were generated by analysing the normalised RNAseq data generated for eight life-stages as part of the *G. pallida* genome project (Cotton *et al*., 2014). Expression was calculated as a logarithm in base 10 of the normalised number of reads for a specific transcript in each developmental stage. J2 here refers to pre-parasitic J2. (G) Evaluation by qRT-PCR of *GpIA7* expression in eggs, pre-parasitic J2s (J2), parasitic J2 (5 days post infection (dpi)) and

young females at 14-21 dpi (y. females). The primer pair used amplifies both paralogue sequences. Expression was calculated relative to that in eggs (using *GpEF1α* for normalisation). Root segments of pouch-grown plantlets infected with *G. pallida* were collected at 5 dpi. The roots were thoroughly rinsed prior to collection in order to eliminate any pre-parasitic J2. Young females were collected from infected roots as described Thorpe *et al*. (2014). The results represent the mean of at least 5 biological replicates each with three technical replicates. Values are means and error bars represent standard error of the mean.

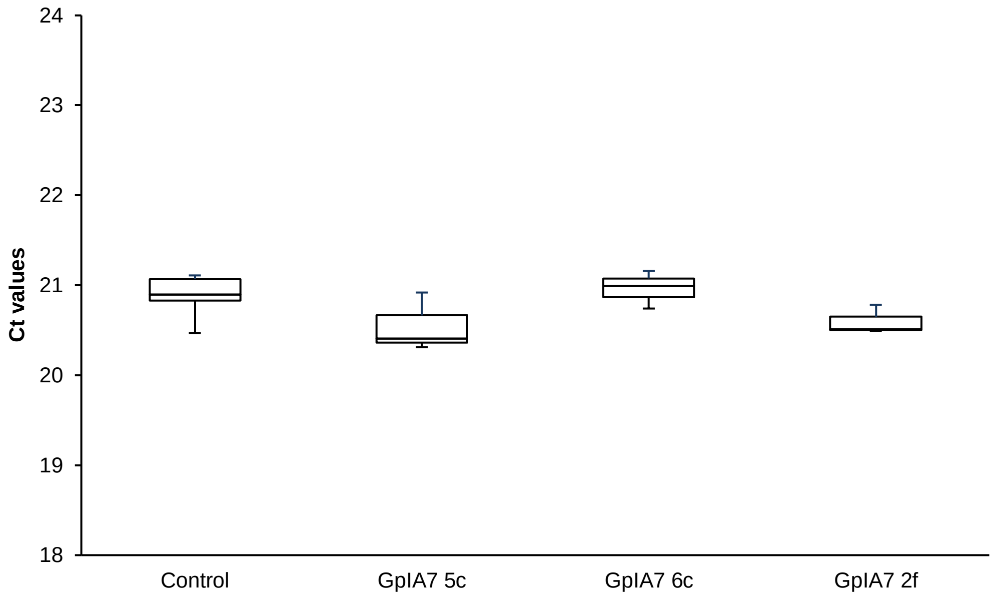

Fig. S2 Validation of *StEF1-α* as a reference gene for qRT-PCR analysis in this study. Expression of the *StEF1-α* reference gene is shown as Ct values determined by qRT-PCR using equal quantities of cDNA from the mock-transformed control potato plants and the GpIA7 transgenic lines. Box plots represent the interquartile range (IQR, 25th–75th). Horizontal bars represent the median. Whiskers indicate the minimum and maximum values (n≥3).

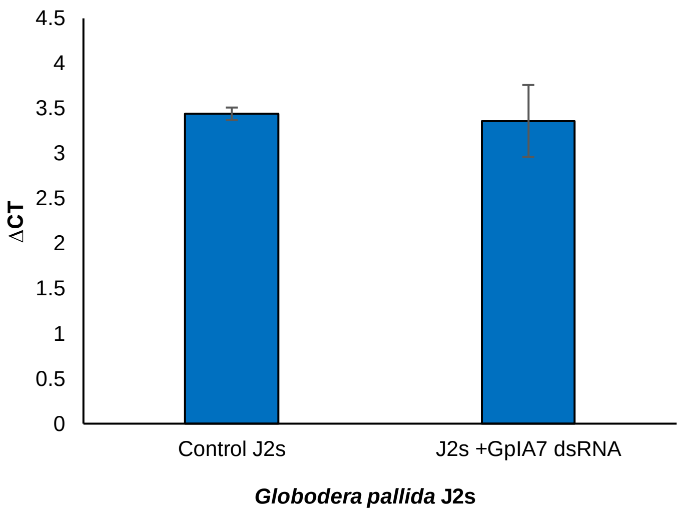

Fig. S3 Evaluation by qRT-PCR of *GpIA7* expression in *Globodera pallida* J2s treated with GpIA7 dsRNA. Expression is presented as a difference in threshold cycle (ΔCT) between the reference gene, *GpEF1α,* and *GpIA7*. The *GpIA7* transcripts are not down-regulated following the treatment of the J2s with dsRNA. The results represent the means of three independent experiments, each with three technical replicates. Error bars are standard error of the mean.

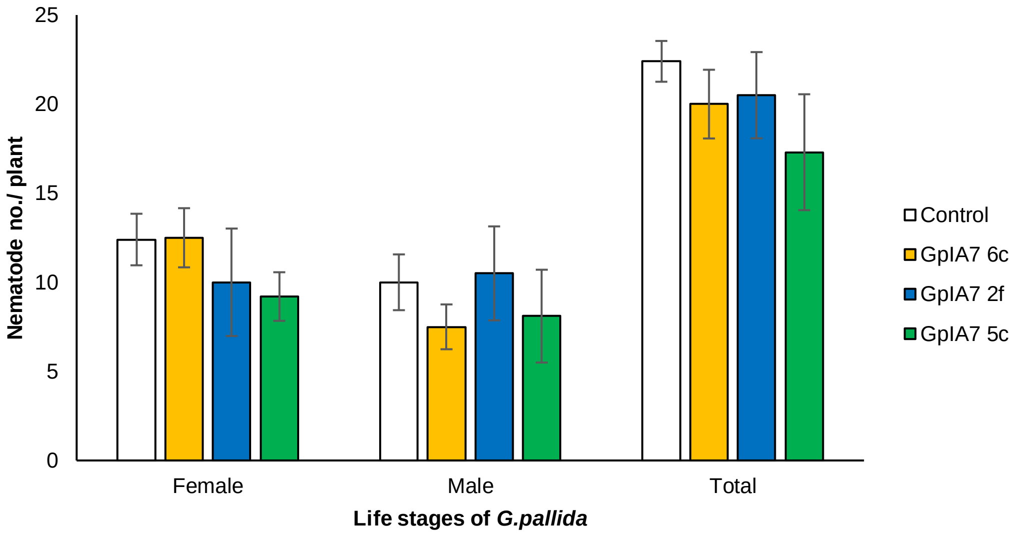

Fig. S4 Evaluation of the susceptibility of GpIA7 transgenic overexpression potato lines to *Globodera pallida.* Plants of GpIA7 potato lines 5c, 6c and 2f were grown in pouches and infected with J2s of *G. pallida*. After 17 d, the roots were stained and the number of worms per root system as well as their developmental stages were recorded. There was no significant difference in the total number of nematodes counted or the life stages observed between the GpIA7 lines and the mock-transformed control plants. Values are means and the error bars are standard error of the mean (*n*=10) (ANOVA).

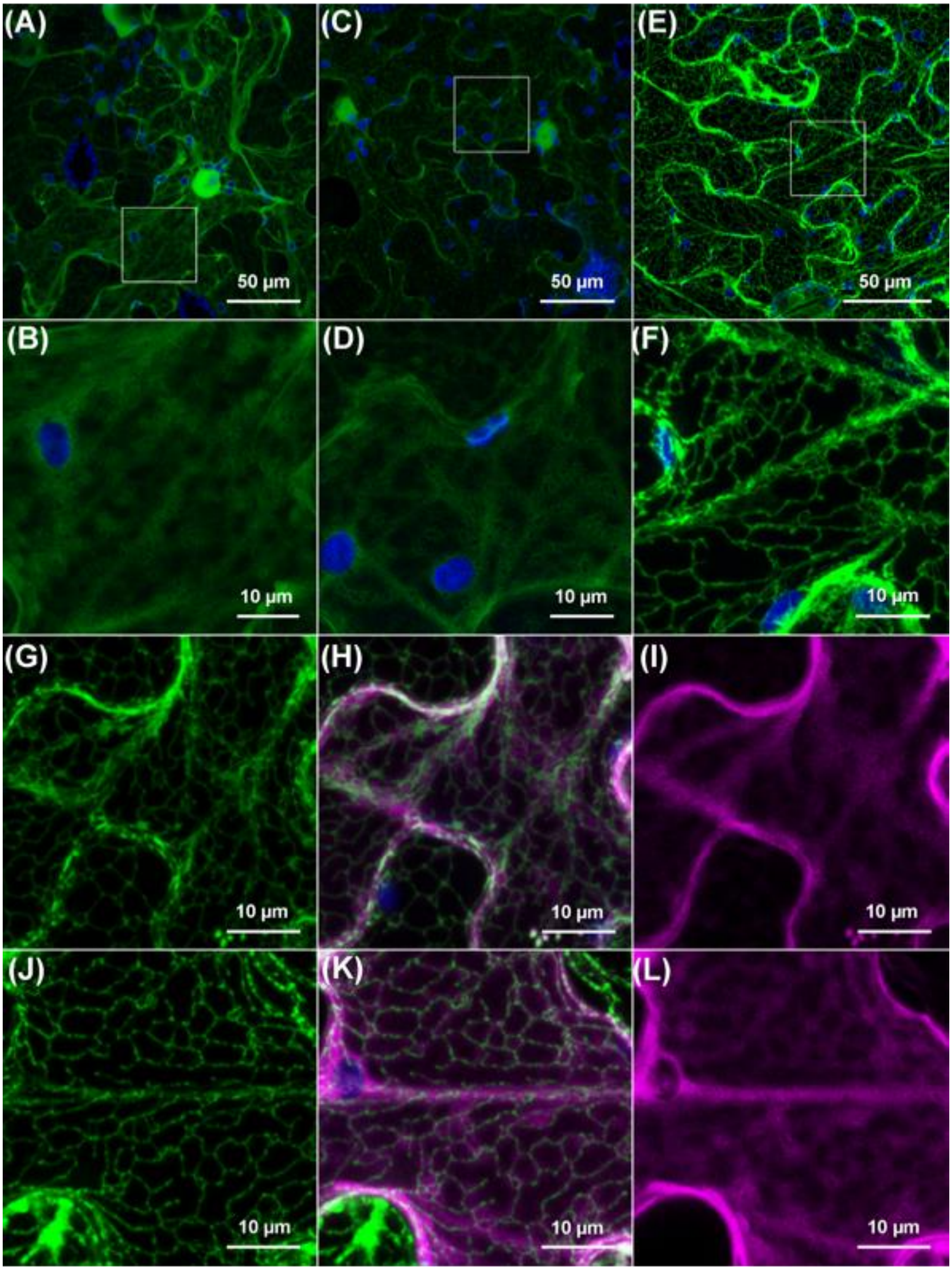
(A)
50 µm
(C)
50 µm
(E)
50 µm
(B)
10 µm
(D)
10 µm
(F)
10 µm
(G)
10 µm
(H)
10 µm
(I)
10 µm
(J)
10 µm
(K)
10 µm
(L)
10 µm

(M)

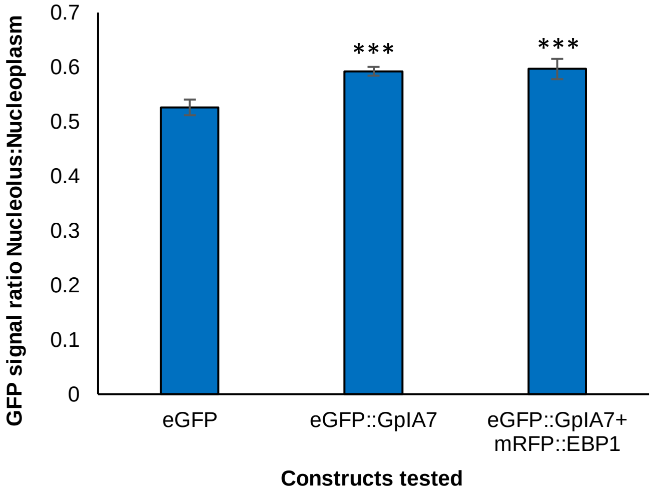

Fig. S5 Subcellular localisation of GpIA7 and StEBP1 ***in planta*****. Assessment of the nucleolus:nucleoplasm partitioning of GpIA7 in plant cell nuclei.** (A-L) *Agrobacterium*-mediated transient expression of *GpIA7* (A,B) lacking its endogenous signal peptide and tagged with the enhanced green fluorescent protein (eGFP) as *eGFP::GpIA7* fusion in *N. benthamiana* leaves and free eGFP protein (C,D) both showing the cytoplasmic localisation of GFP, compared to transgenic plants expressing m-gfp5-ER (E,F) with GFP in the endoplasmic reticulum (Ruiz *et al.*, 1998). Transient expression in transgenic plants expressing m-gfp5-ER and RFP::EBP1 (G-I) or RFP::IA7 (J-L) showing the cytoplasmic localisation of RFP. Pictures were taken 2 days post infiltration by confocal microscopy, with GFP, RFP and autofluorescence from chloroplasts displayed in green, magenta and blue, respectively. (A,C,E) are maximum intensity projection images and (B,D,F-L) are regions presented at higher magnification. Each localisation experiment was replicated at least twice. (M) Assessment of the nucleolus:nucleoplasm partitioning of GpIA7 in plant cell nuclei. The eGFP::GpIA7 fusion construct was transiently expressed in *Nicotiana benthamiana* leaves, either alone or in combination with the mRFP::StEBP1 construct; free eGFP localisation was used as comparison. Confocal images were recorded 48h post inoculation for 3 independent biological samples per inoculation. For each

sample, the GFP fluorescence from 20 individual nuclei was collected as z-stacks using a Zeiss LSM710 confocal microscope and the median section through the nucleolus selected. The fluorescence intensity of a standard area within each nucleolus and nucleoplasm was quantified using ImageJ2 (Rueden *et al.,* 2017) allowing calculation of the GFP nucleolus:nucleoplasm signal ratio. Values are means and the error bars are standard error of the mean (*n*=60) with significant statistical differences indicated relative to the free eGFP control (***-*P*<0.0001) (ANOVA, followed by *post hoc* HSD Tukey test). Reference: **Rueden, C.T., Schindelin, J., Hiner, M.C., DeZonia, B.E., Walter, A.E., Arena, E.T., Eliceiri, K.W.** 2017 ImageJ2: ImageJ for the next generation of scientific image data. BMC Bioinformatics 18, Article 529 [doi:10.1186/s12859-017-1934-z] ; **Ruiz, M. T., Voinnet, O., & Baulcombe, D. C.** 1998. Initiation and maintenance of virus-induced gene silencing. The Plant Cell, 10(6), 937–946. https://doi.org/10.1105/tpc.10.6.937

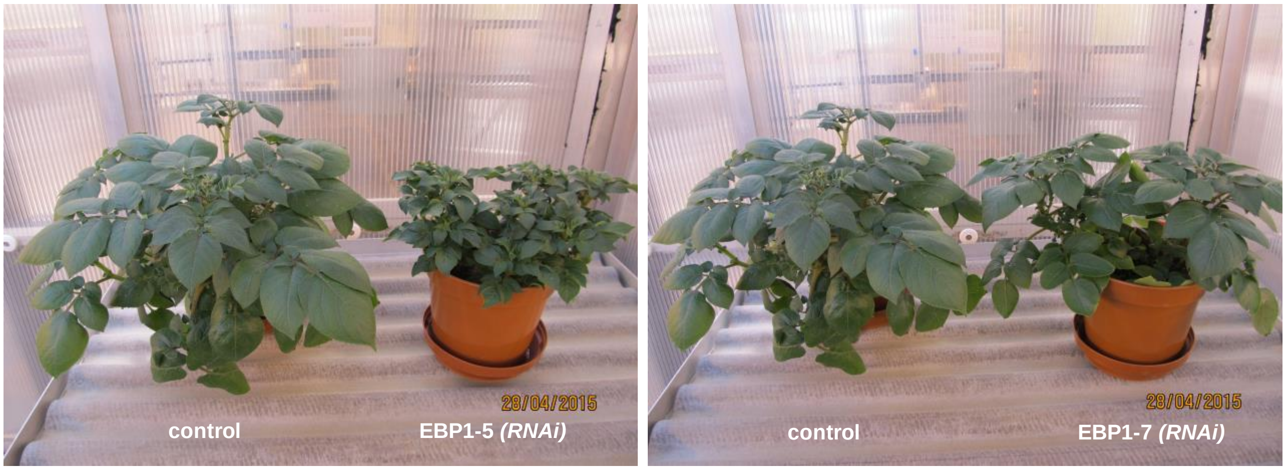

Fig. S6 Downregulation of *StEBP1* through RNAi significantly alters the growth of the transgenic plants. The plant height and leaf size of both EBP1 *(RNAi)* lines is significantly decreased compared to the control plants, with the EBP1-5 *(RNAi)* line showing a more severe phenotype.

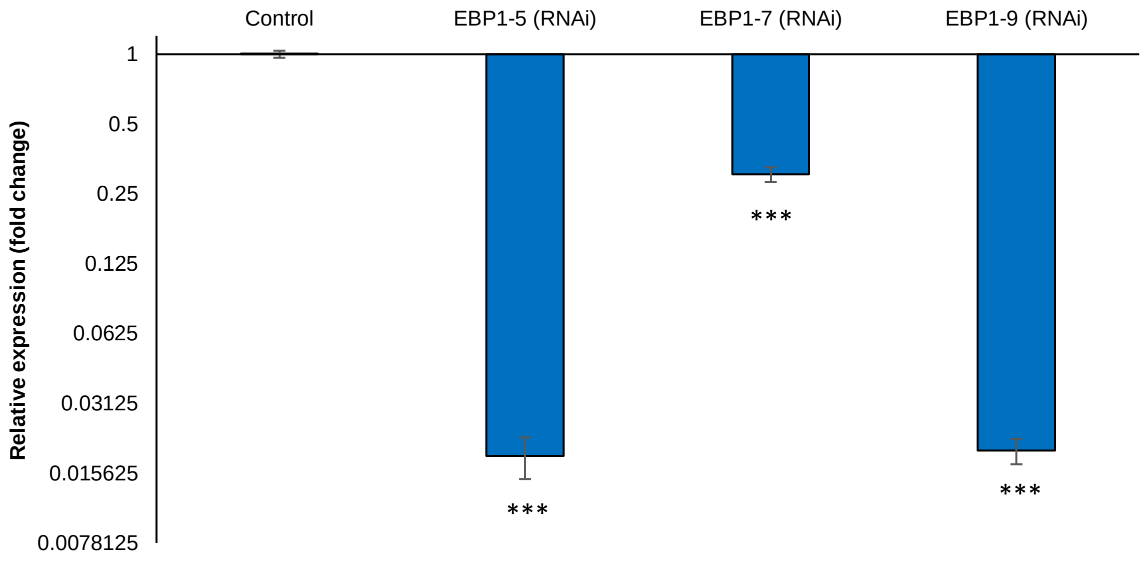

Fig. S7 Evaluation of *StEBP1* gene silencing in the EBP1 *(RNAi*) potato lines. The expression level of *StEBP1* was determined by qRT-PCR in the leaves of three EBP1 *(RNAi)* lines. The fold change in expression was calculated as a ratio between the relative expression (using *StEF1α* for normalisation) of *StEBP1* in EBP1 *(RNAi)* lines and the control plants. At least two biological replicates and three technical replicates were used. Values are mean and error bars represent standard error of the mean. (*** *P*<0.001) (ANOVA, followed by Bonferroni *post hoc* correction)

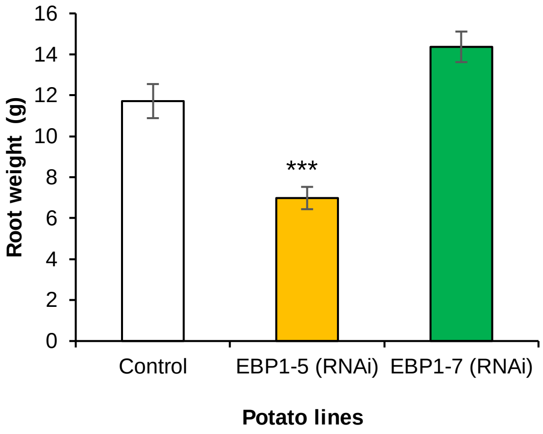

Fig. S8 Evaluation of the root mass of EBP1 *(RNAi)* potato lines. The root mass of EBP1-5 (RNAi) line but not EBP1-7 was significantly reduced compare to the root mass of the control plants. Values are means and error bars represent standard error of the mean; (n=8) (*** P<0.001) (ANOVA, followed by Bonferroni *post hoc* correction).

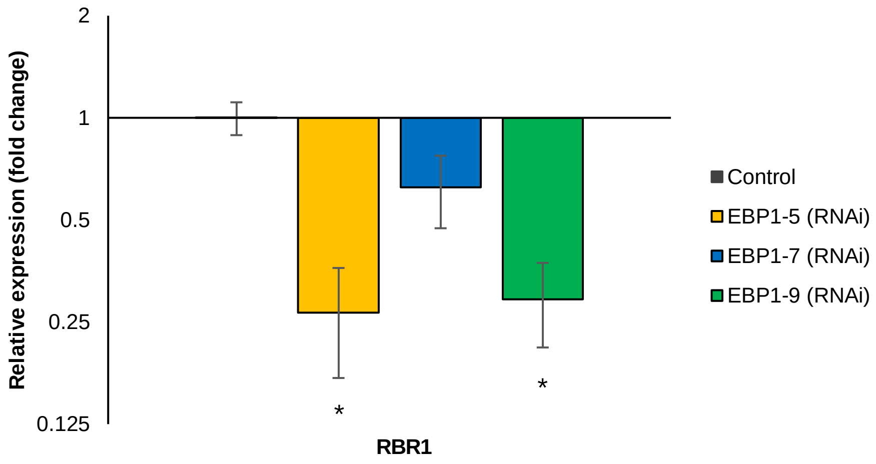

Fig. S9 Relative expression of the *StRBR1* gene in EBP1 *(RNAi)* potato lines. The expression level of *StRBR1* was determined by qRT-PCR in the leaves of three independent EBP1 *(RNAi)* lines. The fold change in expression was calculated as a ratio between the relative expression (using *StEF1α* for normalisation) of the EBP1 *(RNAi)* lines and the control line. At least two biological replicates and three technical replicates were used. (* P<0.05) (ANOVA, followed by Bonferroni *post hoc* test)

Table S1 Primers used in this study

**Gene**	**Primers**
**Cloning experiments**	
GpIA7 (GPLIN_000638300)	
GpIA7 forward (including leader sequence)	ACCATGCAGGACGCTGCTCCCAT
GpIA7 reverse	TCAGCAAAACTTGCAGGTTTTTGG
EBP1 (PGSC0003DMG400030365; NM_001288259)	
EBP1 forward	ATGTCGGACGACGAGAGAGAAG
EBP1 reverse	CTATCCTTCCATAGGCTCAGC
Gp4D06 (GPLIN_000243800)	
Gp4D06 forward (including leader sequence)	ACCATGGCCCCGCATCCATGC
Gp4D06 reverse	GTTGGCGGCGCTGTATTT
Gp16H02 (GPLIN_000854400)	
Gp16H02 forward (including leader sequence)	ACCATGCAATTACAATCGAAGAGCATCGG
Gp16H02 reverse	CAAAAGGCGAAAGCACCG
**YTH cloning experiments**	
EBP1 (PGSC0003DMG400030365; NM_001288259)	
YTH EBP1 forward	GGAGGCCAGTGAATTCATGTCGGACGACGAGAGAG
YTH EBP1 reverse	CGAGCTCGATGGATCCCTATCCTTCCATAGGCTCAGC
GpIA7 (GPLIN_000638300)	
YTH GpIA7 forward	CATGGAGGCCGAATTCCAGGACGCTGCTCCCATCACC
YTH GpIA7 reverse	GCAGGTCGACGGATCCTCAGCAAAACTTGCAGGTTTTTG

Gp4DO6 (GPLIN_000243800)	
YTH Gp4DO6 forward	CATGGAGGCCGAATTCGCCCCGCATCCATGCTGTCC
YTH Gp4DO6 reverse	GCAGGTCGACGGATCCTCAGTTGGCGGCGCTGTATTT
Gp16H02 (GPLIN_000854400)	
YTH Gp16H02 forward	CATGGAGGCCGAATTCCAATTACAATCGAAGAGCATCG
YTH Gp16H02 reverse	GCAGGTCGACGGATCCTCACAAAAGGCGAAAGCACCGA
**Pull down cloning**	
GpIA7 (GPLIN_000638300)	
pQE30 GpIA7 forward	ACAGGATCCCAGGACGCTGCTCCCATC
pQE30 GpIA7 reverse	ACAAAGCTTCAGCAAAACTTGCAGGTT
EBP1 (PGSC0003DMG400030365; NM_001288259)	
pBI EBP1 forward	ACATCTAGATCGGACGACGAGAGAGAA
pBI EBP1 forward	ACAGGTACCCTATCCTTCCATAGGCTC
***In situ* hybridization experiments**	
GpIA7 (GPLIN_000638300)	
GpIA7 forward	AGGACGCTGCTCCCATCACC
GpIA7 reverse	GCAGGTTTTTGGGCACATCG
**qRT-PCR experiments**	
GpIA7 (GPLIN_000638300)	
GpIA7 forward	GTCCTCAAGCTGTACCGACC
GpIA7 reverse	CTTGCAGGTTTTTGGGCACA
GpIA7 (GPLIN_000638300)	
GpIA7 forward (RNAi)	TGCGTTTTGTTGCTGATTTC
GpIA7 reverse (RNAi)	GGTCGGTACAGCTTGAGGAC
StEF1 alpha (AB061263)	
EF1 alpha forward	ATTGGAAACGGATATGCTCCA
EF1 alpha reverse	TCCTTACCTGAACGCCTGTCA
StEBP1 (***NM_001288259***)	
EBP1 qF	GAGCGTGTCCAATCCTGACA
EBP1 qR	TTCCATCACCAGTGCTCGTC

StRNR2 (XM_006338637.2)	
RNR2 forward	TCCGTGCAATCGAGACCATC
RNR2 reverse	TCGGAACCATCGATCCAACG
StCYCD3;1 (PGSC0003DMT400066557 as ortholog of At4g34160***)***	
CYCD3;1 forward	CTTGACTTCCAAGTGGAGGATG
CYCD3;1 reverse	ACAGCACCAGAAGCTCCATTC
StCDKB1;1 (XM_006362266.2)	
CDKB1;1 forward	TGCCCTCATCCAGAGTTTCT
CDKB1;1 reverse	GCGGCTTCAAATCTCTGTGG
StRBR1 (XM_006340861)	
RBR1 forward	TCGACGCAGATGGTTTGACC
RBR1 reverse	GACCCCAGCAAGCTATCCTC
**RNAi experiments**	
GpIA7 RNAi F *Xba*I	ACATCTAGACAGGACGCTGCTCCCATC
GpIA7 RNAi R *Xho*I	ACACTCGAGGCAAAACTTGCAGGTTTTTG
EBP1 sense F *Xho*I	ACACTCGAGCTGGTGATGGAAAGCCCAAGTTG
EBP1 sense R *Kpn*I	ACAGGTACCTCCATAGGCTCAGCTTGAGATG
EBP1 anti-sense F *Xba*I	ACATCTAGACTGGTGATGGAAAGCCCAAGTTG
EBP1 anti-sense R *Cla*I	ACAATCGATTCCATAGGCTCAGCTTGAGATG
**Sequencing experiments**	
M13 forward (-20)	GTAAAACGACGGCCAG
M13 reverse	CAGGAAACAGCTATGAC
T7 promoter forward	TAATACGACTCACTATAGGG
pCL112-NYFP-F	CAACTACAACAGCCACAACG
pCL113-CYFP-F	CCGACAACCACTACCTGAG
